# HMGA1 regulates the Plasminogen activation system in the secretome of breast cancer cells

**DOI:** 10.1038/s41598-017-11409-4

**Published:** 2017-09-18

**Authors:** Giulia Resmini, Serena Rizzo, Cinzia Franchin, Rossella Zanin, Carlotta Penzo, Silvia Pegoraro, Yari Ciani, Silvano Piazza, Giorgio Arrigoni, Riccardo Sgarra, Guidalberto Manfioletti

**Affiliations:** 1 Department of Life Sciences, University of Trieste, Trieste, Italy; 2 Department of Biomedical Sciences, University of Padova, Padova, Italy; 3 Proteomics Center, University of Padova and Azienda Ospedaliera di Padova, Padova, Italy; 4 National Laboratory CIB (LNCIB), Area Science Park, Trieste, Italy; 5 Present Address: I.R.C.C.S. Policlinico San Donato, Milano, Italy; 6 Present Address: Bioinformatics Core Facility, Centre for Integrative Biology, CIBIO, University of Trento, Trento, Italy

## Abstract

Cancer cells secrete proteins that modify the extracellular environment acting as autocrine and paracrine stimulatory factors and have a relevant role in cancer progression. The HMGA1 oncofetal protein has a prominent role in controlling the expression of an articulated set of genes involved in various aspect of cancer cell transformation. However, little is known about its role in influencing the secretome of cancer cells. Performing an iTRAQ LC–MS/MS screening for the identification of secreted proteins, in an inducible model of HMGA1 silencing in breast cancer cells, we found that HMGA1 has a profound impact on cancer cell secretome. We demonstrated that the pool of HMGA1–linked secreted proteins has pro–migratory and pro-invasive stimulatory roles. From an inspection of the HMGA1–dependent secreted factors it turned out that HMGA1 influences the presence in the extra cellular *milieu* of key components of the Plasminogen activation system (PLAU, SERPINE1, and PLAUR) that has a prominent role in promoting metastasis, and that HMGA1 has a direct role in regulating the transcription of two of them, i.e. PLAU and SERPINE1. The ability of HMGA1 to regulate the plasminogen activator system may constitute an important mechanism by which HMGA1 promotes cancer progression.

## Introduction

Cancer remains one of the major devastating diseases throughout the world. In particular, breast cancer (BC) is one of the leading causes of cancer-related deaths in women. Mortality from BC is mainly due to distant metastasis, therefore there is an urgent need to identify molecular networks early involved in conferring cells the ability to migrate and escape their original residency site. Breast cancer is extremely heterogeneous and several different deregulated factors have been demonstrated as possible driver of cancer onset. HMGA1 overexpression has a prominent role in breast cancer progression by reprogramming cancer cells to a stem-like state and conferring them aggressiveness, both in term of cell migration, invasion, and metastatic capabilities^1–5^.

HMGA1 protein is an oncofetal architectural transcription factor that constitutes a critical hub in the chromatin network^6^ and has a causal role in neoplastic transformation^7^. More importantly, from a clinical point of view, high expression levels of HMGA1 in cancer specimens portend a poor prognosis in several tumors^8^ among which breast cancer. We recently demonstrated that in Triple Negative Breast Cancer (TNBC) cells the silencing of HMGA1 leads to the reversion of cancer–related phenotypes, such as mesenchymal to epithelial transition (MET), migration and invasion *in vitro*, and the formation of metastases *in vivo*, due to many transcriptome and proteome alterations^3–5^.

With the aim of unravelling novel cancer–related mechanisms exploited by HMGA1 we focused our study on secreted proteins (SPs) since they represent an important protein category that has a fundamental role in driving cancer progression^9^. Indeed SPs modify the extra cellular milieu, activate/recruit host cells, and are part of the autocrine and paracrine cancer signalling system^9^.

The term “secretome” is referred to the complex set of molecules secreted from living cells via the classical, the non-classical, or the exosome pathways^10^. Culture media (CM) have been shown to be a valuable resource for secretomic studies since SPs released in the medium reflect the actual state of the cultured cells. However, analysis of SPs in CM is quite challenging; this is attributed to technical difficulties given by (i) low protein concentrations, (ii) masking and contamination by intracellular proteins released due to cell lysis or death, and (iii) masking by high abundant proteins, such as albumin, present in CM^11^. These limits are overcome (i) by managing a high number of cells, (ii) by adopting affinity purification strategies, and (iii) by working in serum starvation conditions.

In the present work, we exploited a lectin affinity chromatography to enrich SPs from serum starved HMGA1-expressing and HMGA1-silenced MDA-MB-231 cells. SPs obtained from HMGA1-expressing cells turned out to have an enhanced effect in stimulating cell migration. By an isobaric Tag for Relative and Absolute Quantitation liquid chromatography–tandem mass spectrometry (iTRAQ LC–MS/MS) differential screening we identified SPs whose abundance was linked to HMGA1 expression level. These SPs were evaluated for their prognostic value with respect to relapse free survival (RFS) and distant-metastasis free survival (DMSF) and among the proteins with a prognostic value we searched those transcriptionally regulated by HMGA1. For the first time we demonstrated that HMGA1 has a direct role in regulating PLAU and SERPINE transcription thus directly influencing the urokinase plasminogen activator system, which is one of the best-characterized pathways involved in the metastatic spreading of cancer cells.

## Results

**Figure 1:**
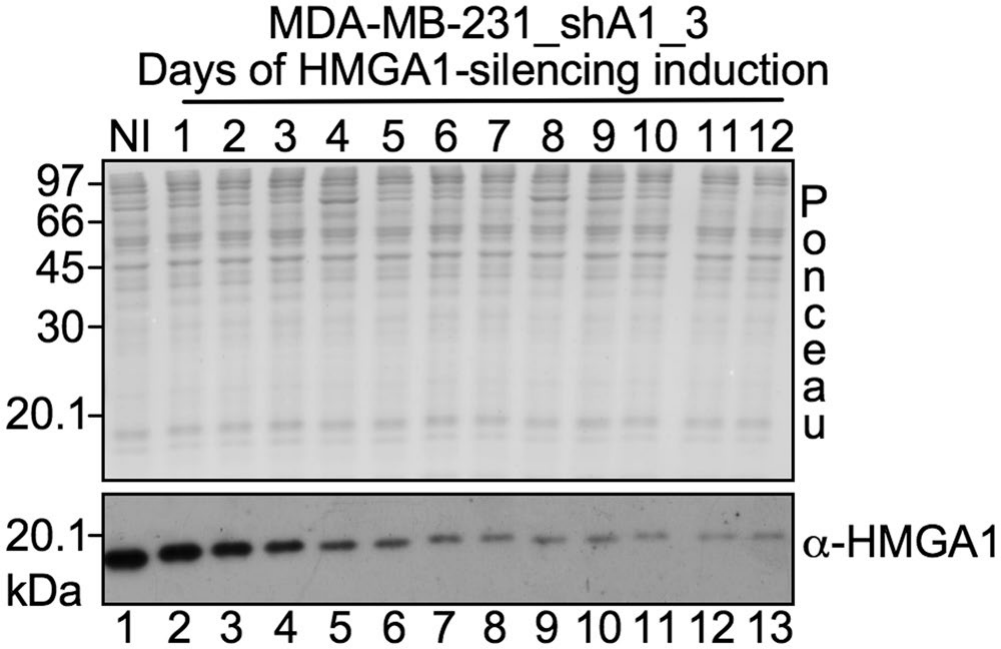
HMGA1 silencing in MDA-MB-231_shA1_3 clone. Western blot analysis of total protein lysates of MDA-MB-231_shA1_3 cells. Proteins were SDS–PAGE analysed, transferred on nitrocellulose membrane, and stained with Ponceau Red. The expression of HMGA1 was detected by anti-HMGA1 antibody. The MDA-MB-231_shA1_3 clone was induced for the silencing of HMGA1 with doxycycline for 1 to 12 days (lanes 2–13); as a control, not induced (NI) cells are shown in lane 1. Molecular weight markers (kDa) are shown on the left. The red ponceau stained membrane is shown to verify the protein loading normalization.

We developed a model for the reversion of the tumoral phenotype based on the inducible silencing of the oncofetal HMGA1 protein in the TNBC cell line MDA-MB-231 by shRNA. Upon HMGA1 down–regulation, cells change morphology (more flattened and polygonal), grow as an ordered epithelial monolayer sheet, and lose invasive and metastatic properties^3^. HMGA1 silencing reached its maximum effect after 6 days and then essentially stays level (Fig. 1).

### Assessing the reliability of a lectin-affinity chromatographic method for the purification of secreted proteins from serum starved cells

HMGA1 has a driving role in breast cancer development, however, its contribution in modulating the abundance of proteins acting in the extra–cellular milieu and/or involved in cell/cell communication is still almost unexplored.

The study of SPs is challenging for three main limitations: (i) the low abundance of SPs; (ii) serum proteins of culture media mask SPs; (iii) proteins released from dead cells mask SPs as well. Collecting high CM volumes and adopting ultrafiltration procedures to concentrate SPs solve the first limitation. Purifying SPs from serum starved cell cultures solves the second problem while the third limitation is solved by adopting an affinity purification step to enrich for glycosylated proteins, a post-translational modification peculiar for proteins exposed to the extra–cellular environment.

Serum starvation represents a stress condition for cells and therefore to check whether MDA-MB-231 shA1_3 cells induced (I) or not induced (NI) for the silencing of HMGA1 (that for simplicity hereinafter we will call shA1_3 I and shA1_3 NI, respectively) suffer this treatment, we evaluated their serum starved growth in comparison with a normal condition. In Supplementary Fig. 1a it is shown that MDA-MB-231 and shA1_3 I and NI cells continue to grow also when serum starved, albeit with a lower rate and that at 30 h after serum starvation (time window chosen for Conditioned CM (CCM) collection) cells are still in a rising phase of growth.

To evaluate the efficiency of lectin (ConA – concanavalin A – and WGA – wheat germ agglutinin –) affinity chromatographic enrichment we tested it with untreated control (Ctrl) MDA-MB-231 CCM. CCM was incubated with the mixture of agarose bound lectins ConA/WGA or unbound agarose (negative control) and the captured glycoproteins (GPs) were eluted by competition with methyl-α-D-mannopyroside and N-acetyl-glucosamine. The lectin-based approach allows the isolation of a specific subset of proteins being negligible the amount of those unspecifically bound to the resin (Supplementary Fig. 1b). We performed an MS-based evaluation of the enriched proteins. A 1D-SDS PAGE lane, in which purified GPs were separated, was cut in ten gel slices and each of them was subjected to in gel digestion and LC-MS/MS analysis. We identified a total of 220 proteins (Supplementary Table 1) and bioinformatic analyses revealed that most of them were glycosylated (Supplementary Fig. 1c). These data confirmed the efficiency of our purification strategy.

### Secreted glycoproteins from HMGA1 expressing MDA-MB-231 cells promote cell motility

As mentioned above, MDA-MB-231 cells in which HMGA1 expression is abrogated loose their migratory and invasive properties. In order to verify whether these properties are at least in part regulated by HMGA1-dependent SPs, we evaluated the effect of affinity purified GPs obtained from shA1_3 I and NI cells on the proliferation and migratory properties of the MDA-MB-468 breast cancer cell line, a TNBC cell line that can be used as suitable model for both EMT and MET^12,13^.

**Figure 2:**
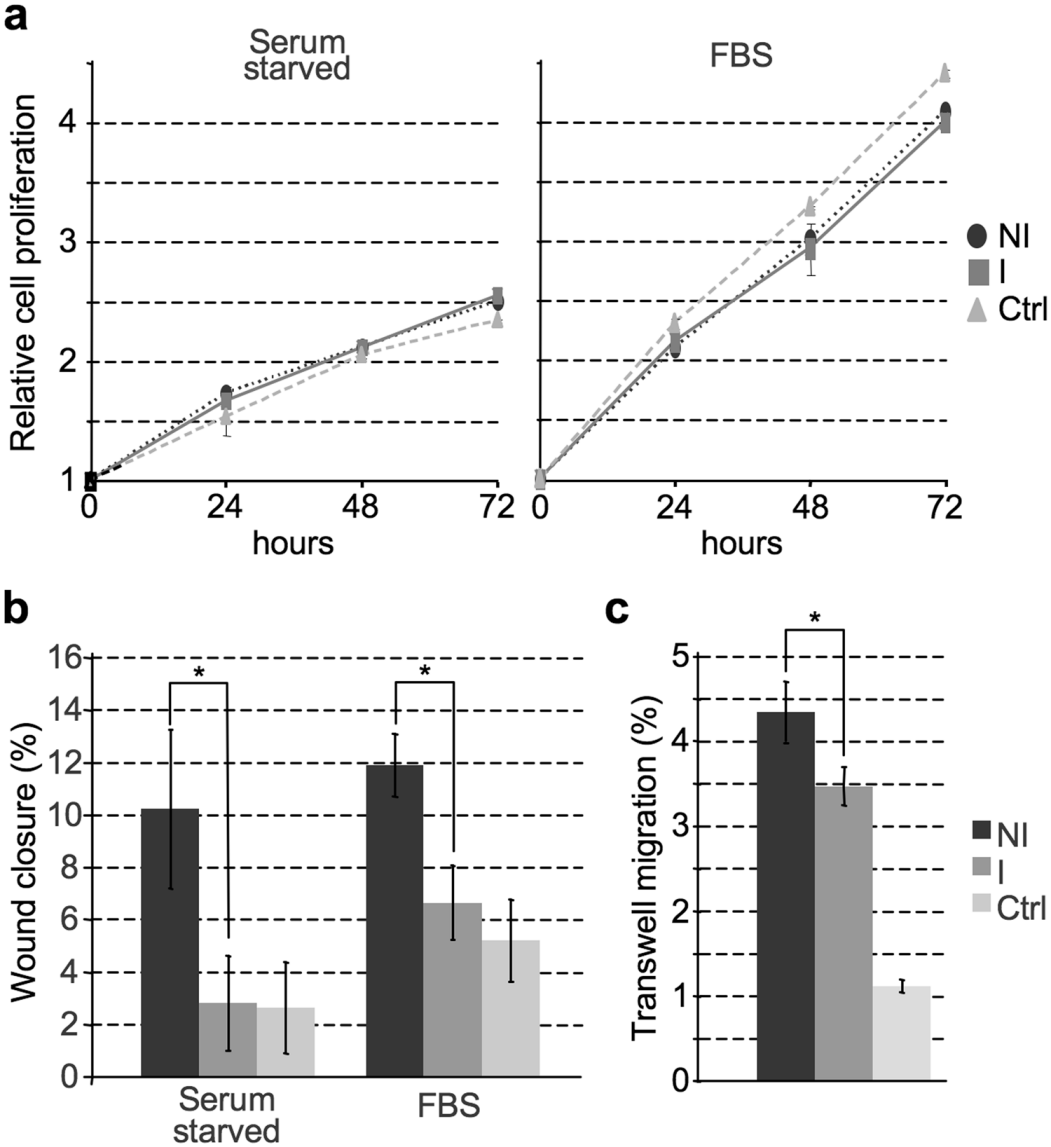
Secreted glycoproteins obtained from HMGA1 expressing MDA–MB–231 shA1_3 cells positively influence cancer cells motility. MDA-MB-468 cells were plated and treated with glycoproteins (GPs) purified from CM of MDA-MB-231_shA1_3 cells induced (I) or not induced (NI) for HMGA1 silencing. The quantity of GPs used in each well corresponds to 0.35 µg for MTS assay, 3.5 µg for wound healing assay, 1.31 µg for transwell assay. Control experiments (Ctrl) were performed without the addition of any GPs. (**a**) MTS assays were performed at 0, 24, 48, and 72 h both in the presence of FBS and in a serum starved condition. Values are averages ± SD (n = 3). The metabolic activity of cells at starting point (0 h) obtained by the MTS assay was arbitrary set to 1. (**b**) Wound healing assays were performed measuring wound width at 0 and 24 h, both in the presence of FBS and in a serum starved condition. Values are means ± SD (n = 4). (**c**) Transwell assays were performed counting migrated cells after 24 h. Values are average ± SD (n = 4). Statistical significance was assessed with Student’s t-test (*P < 0.05).

We evaluated cell proliferation by MTS assay and cell migration by wound healing and transwell assays. When possible, we tested both serum-starved cells and cells growing in complete medium. As shown in Fig. 2a, treatment of MDA-MB-468 cells with GPs (equivalent amount in terms of μg) obtained from shA1_3 NI and I cells does not confer a relevant change in cell proliferation with respect to control untreated cells (Ctrl). On the contrary cell migration is affected. Indeed, GPs obtained from shA1_3 NI cells promote MDA-MB-468 cells migration both in wound healing and transwell assays (panels b and c) with a significative difference with respect to cells treated with GPs obtained from shA1_3 I cells (i.e. cells in which the expression level of HMGA1 has been silenced) and to mock treated cells (Ctrl). These data suggest that the secretome of HMGA1-expressing MDA-MB-231 cells (shA1_3 NI) includes proteins involved in the modulation of cell migration and that these factors are retained and are still effective after lectin affinity purification.

### iTRAQ analyses for the identification of differentially secreted proteins in HMGA1-expressing and –depleted MDA-MB-231 cells

Considering the different biological activity displayed by GPs obtained from shA1_3 I and shA1_3 NI cells, we decided to perform a quantitative protein comparison based on iTRAQ technology.

GPs have been obtained from serum-starved shA1_3 I and NI cells (biological triplicates), in which HMGA1 silencing has been checked by WB analysis (Supplementary Fig. 2a), and quantified by Lowry method (see Supplementary Fig. 2b for a SDS–PAGE analysis assessing correct protein normalization).

50 μg of purified GPs have been accumulated at the stacking/running interface of a 1D SDS polyacrilamide gel, briefly stained by blue coomassie and the corresponding bands have been cut and in gel digested. Peptides have been recovered, quantified, and subjected to preparation for iTRAQ analysis.

iTRAQ–based comparative proteomic analyses (Supplementary Table 2A and B) provided the identification of a total of 463 proteins, 344 of which were detected in at least two biological replicates.

Ingenuity^®^ Pathway Analysis (IPA^®^) performed on the 344 identified proteins highlights that these proteins are strongly associated with molecular and cellular functions linked to cell motility and proliferation and that the top scoring associated disease is cancer (Table 1). The overlap with the set of proteins identified with the ion trap preliminary experiments is of about 1/3 (131/344). However, also in this case, there is a strong enrichment of secreted and glycosylated proteins (Supplementary Fig. 2c). Considering separately those down (fc < 0.7) and the up (fc > 1.3) regulated (Table 1), it is possible to evidence that the downregulated ones are strongly associated with cell motility and cancer. Noteworthy, these bioinformatic evidences are in full agreement with wound healing and transwell assays, showing that secreted proteins from shA1_3 NI, with respect to those from shA1_3 I cells, contribute in regulating cell motility.

**Table 1:** Bioinformatic analysis (Ingenuity Patway Analysis) of the differentially secreted proteins following HMGA1 silencing.

	Name	p-value of overlap	# molecules
Molecular and Cellular Function			
*ALL*			
1	Cellular Movement	1.47 × 10^−08^–4.24 × 10^−50^	166
2	Cellular growth and Proliferation	3.26 × 10^−08^–2.91 × 10^−36^	191
3	Cell Death and Survival	3.47 × 10^−08^–1.31 × 10^−34^	190
4	Cell-To-Cell Signaling and Interaction	2.99 × 10^−08^–1.82 × 10^−33^	140
5	Cell Morphology	3.49 × 10^−08^–2.17 × 10^−28^	165
*DOWN REGULATED* (<*0.7*)			
1	Cellular Movement	6.89 × 10^−03^–6.68 × 10^−08^	15
2	Post-translational modification	5.65 × 10^−03^–1.68 × 10^−07^	9
3	Protein Degradation	1.13 × 10^−03^–1.68 × 10^−07^	8
4	Protein Synthesis	4.45 × 10^−03^–1.68 × 10^−07^	8
5	Cell Morphology	5.65 × 10^−03^–5.71 × 10^−07^	12
*UP REGULATED* (>*1.3*)			
1	Cellular growth and Proliferation	6.89 × 10^−03^–5.02 × 10^−08^	26
2	Cellular Movement	7.66 × 10^−03^–2.54 × 10^−07^	19
3	Cell Morphology	7.66 × 10^−03^–1.42 × 10^−06^	19
4	Cell-To-Cell Signaling and Interaction	7.66 × 10^−03^–7.9 × 10^−06^	15
5	Carbohydrate Metabolism	3.83 × 10^−03^–8.49 × 10^−06^	9
**Diseases and Disorders**			
*ALL*			
1	Cancer	3.58 × 10^−08^–3.16 × 10^−36^	313
2	Organismal Injury and Abnormalities	3.34 × 10^−08^–3.16 × 10^−36^	315
3	Inflammatory Response	3.62 × 10^−08^–6.03 × 10^−25^	152
4	Developmental Disorders	2.87 × 10^−09^–2.96 × 10^−22^	97
5	Hereditary Disorders	1.12 × 10^−09^–2.96 × 10^−22^	66
*DOWN REGULATED* (<*0.7*)			
1	Cancer	7.06 × 10^−03^–7.07 × 10^−08^	19
2	Connective Tissue Disorders	7.06 × 10^−03^–7.07 × 10^−08^	7
3	Organismal Injury and Abnormalities	7.06 × 10^−03^–7.07 × 10^−08^	19
4	Skeletal and Muscular Disorders	7.06 × 10^−03^–7.07 × 10^−08^	7
5	Inflammatory Response	6.89 × 10^−03^–5.65 × 10^−07^	14
*UP REGULATED* (>*1.3*)			
1	Dermatological Diseases and Conditions	6.92 × 10^−03^–2.61 × 10^−08^	15
2	Developmental Disorders	5.75 × 10^−03^–2.61 × 10^−08^	15
3	Hereditary Disorders	5.75 × 10^−03^–2.61 × 10^−08^	18
4	Organismal Injury and Abnormalities	7.48 × 10^−03^–2.61 × 10^−08^	35
5	Cancer	7.48 × 10^−03^–3.68 × 10^−08^	35

### Clinical–relevance selection of secreted proteins

Our primary aim was to exploit this cellular model to search for HMGA1–linked secreted molecules influencing cell motility/invasiveness that could be involved in conferring cancer cells metastatic abilities. We focused on the down–regulated proteins (i.e. those strictly linked to cell motility and cancer – see Table 1) deciding to investigate the top 20 down regulated ones.

**Figure 3:**
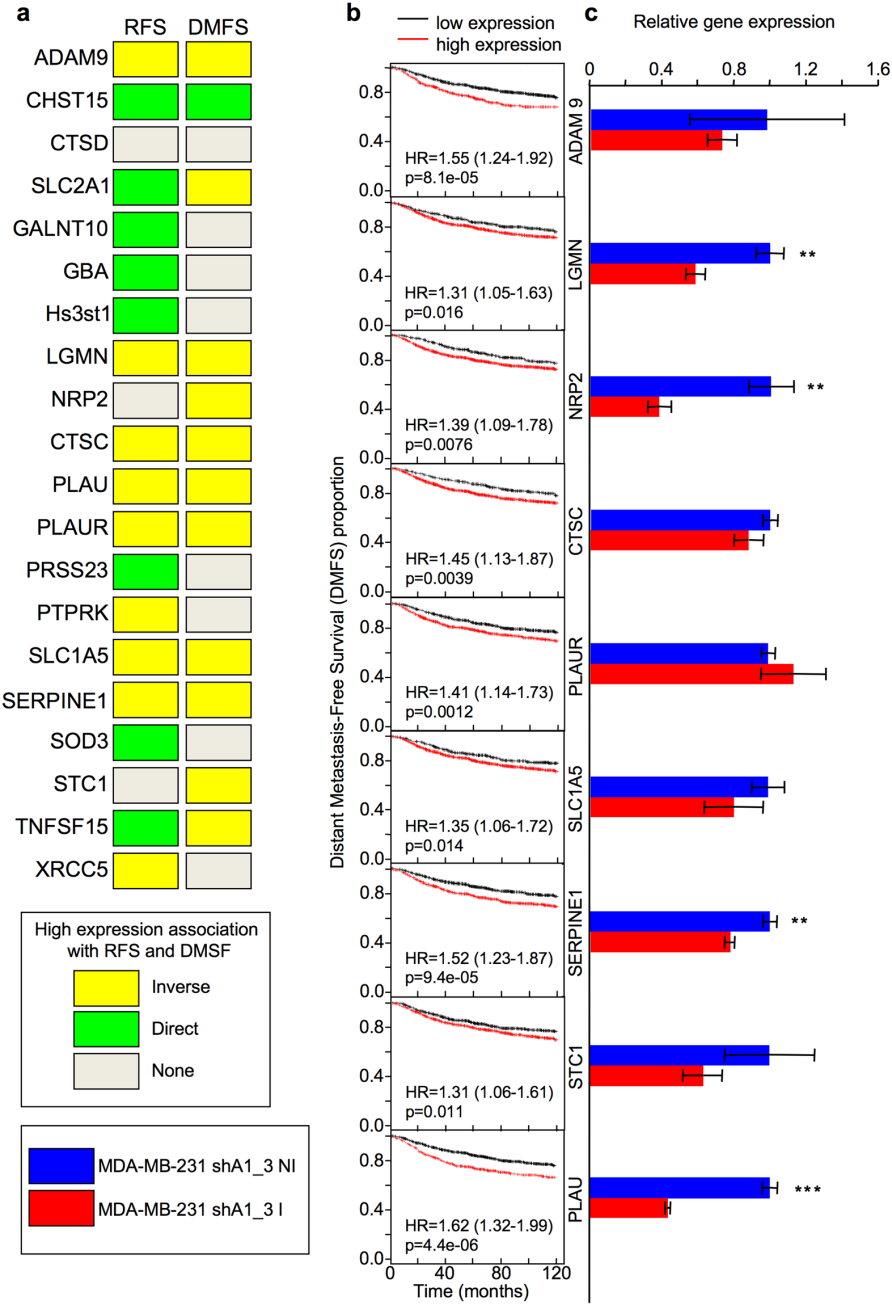
Searching for secreted proteins linked with a worst prognostic value (Relapse and Distant Metastasis Free Survival) whose mRNA is linked to HMGA1 expression levels. (**a**) Summary of data regarding the top 20 down regulated proteins identified in the iTRAQ screening and their prognostic value in term of Relapse and Distant Metastasis Free Survival obtained analysing a breast cancer gene expression meta-dataset. (**b**) DMFS KM plots of those genes whose high expression levels represent a negative prognostic factor for DMFS (p < 0.05) in a breast cancer gene expression meta-dataset. (**c**) The mRNA expression levels of the indicated genes were analysed by qRT-PCR comparing HMGA1-silenced cells (MDA–MB–231_shA1_3 I cells) versus HMGA1 expressing cells (MDA–MB–231_shA1_3 NI cells). GAPDH was used for normalization. Values are means ± SD (n = 3). Statistical significance was assessed with Student’s t-test (**P < 0.01; ***P < 0.001).

We evaluated which of these proteins are linked to a worse prognosis both in terms of relapse– and distant metastasis–free survival. To this end Kaplan–Meier plots were obtained from breast cancer gene expression metadatasets. Comparing low vs. high gene expression levels, 9 proteins (ADAM9, LGMN, NRP, CTCS, PLAUR, SLC1A5, SERPINE1, STC1, and PLAU) turned out to contemporary satisfy both these criteria, thus suggesting that they could be linked to metastatic spread of cancer cells (Fig. 3a).

### HMGA1 regulates the gene expression of a set of differentially secreted proteins

The differential abundance of these proteins in the extracellular *milieu* is linked to HMGA1 expression. However, since we adopted a glycoprotein affinity enrichment on secreted proteins, the observed difference in MDA–MB–231 shA1_3 NI vs. I could be due to several reasons: (i) their expression could be differentially regulated at transcriptional or post-transcriptional level; (ii) their secretion rate could be altered; (iii) their glycosylation levels could be different.

Considering HMGA1 is an architectural transcription factor that has a very profound impact on gene expression regulation^6,14^ we decided to focus on those proteins whose presence in the extra cellular *milieu* could be attributable to a differential transcriptional rate. These proteins could be considered at the base of the HMGA1–dependent pyramidal cascade of events and early involved in tumor cell dissemination. We checked, by qRT-PCR, the gene expression levels of the 9 proteins that displayed a prognostic value in terms of DMFS. The expression of four genes (PLAU, SERPINE1, NRP2, and LGMN) turned out to be significantly downregulated in shA1_3 I cells (Fig. 3b). This result not only evidences that the mRNA expression of a pool of secreted proteins is linked to HMGA1, but also highlights that other mechanisms (as envisioned before) could be perturbed by HMGA1.

### HMGA1-regulated genes have a role in modulating cell motility

**Figure 4:**
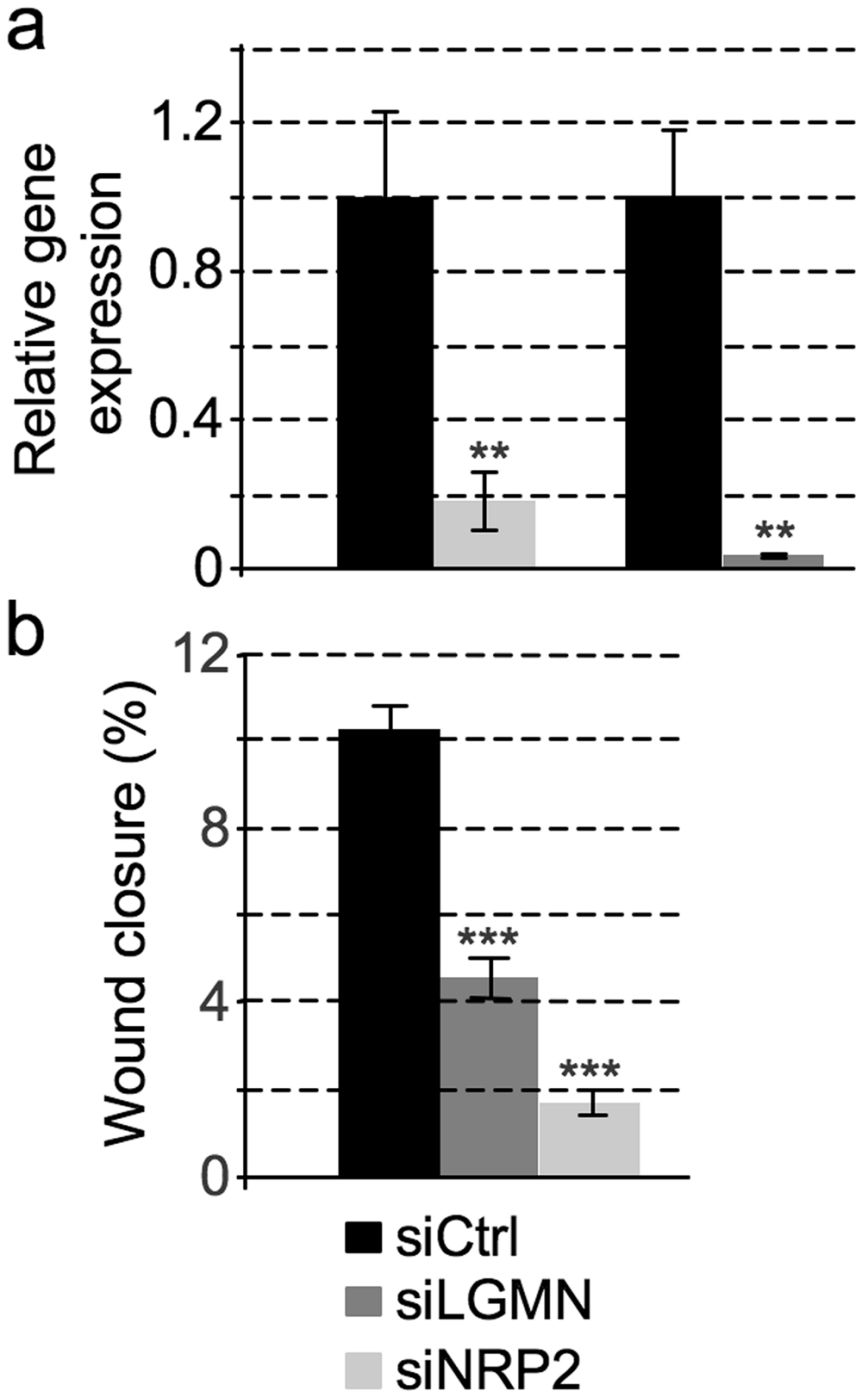
Silencing of Neuropilin 2 (NRP2) and Legumain (LGMN) affects MDA–MB–231 cell motility. MDA–MB–231 cells were treated with siRNA targeting NRP2, LGMN or control siRNA and evaluated for wound closure. (**a**) mRNA expression levels of NRP2 and LGMN were analysed by qRT-PCR comparing NRP2– and LGMN-silenced cells (siNRP2 and siLGMN, respectively) versus control cells (siCtrl). GAPDH was used for normalization. Values are average ± SD (n = 3). (**b**) Wound healing assays were performed to compare cell motility between NRP2 and LGMN silenced and control cells. Values are means ± SD (n = 4). Statistical significance was assessed with Student’s t-test (*P < 0.05; **P < 0.01; ***P < 0.001).

PLAU, SERPINE1, NRP2, and LGMN, are secreted proteins whose mRNA expression is regulated by HMGA1. As concerns SERPINE1 and PLAU, their involvement in modulating breast cancer cell motility and invasiveness is well established^15^, therefore we decided to test the effects on cell motility of the other two (LGMN and NRP2). We silenced LGMN and NRP2 expression in MDA–MB–231 cells and performed wound–healing assays. As can be seen in Fig. 4, the silencing of both factors has an evident negative impact on wound closure. These data further confirm that secreted proteins differentially regulated by HMGA1 (i.e. MDA–MB–231 shA1_3 NI vs. I) have a role in contributing to cell motility.

### HMGA1 directly modulates the transcription of components of the urokinase plasminogen activator system

The urokinase plasminogen activator system is one of the main mechanisms involved in the processes of cell invasion and metastatization. Its activation led both to an extracellular matrix remodelling process and intracellular signalling cascade activation^15,16^.

**Figure 5:**
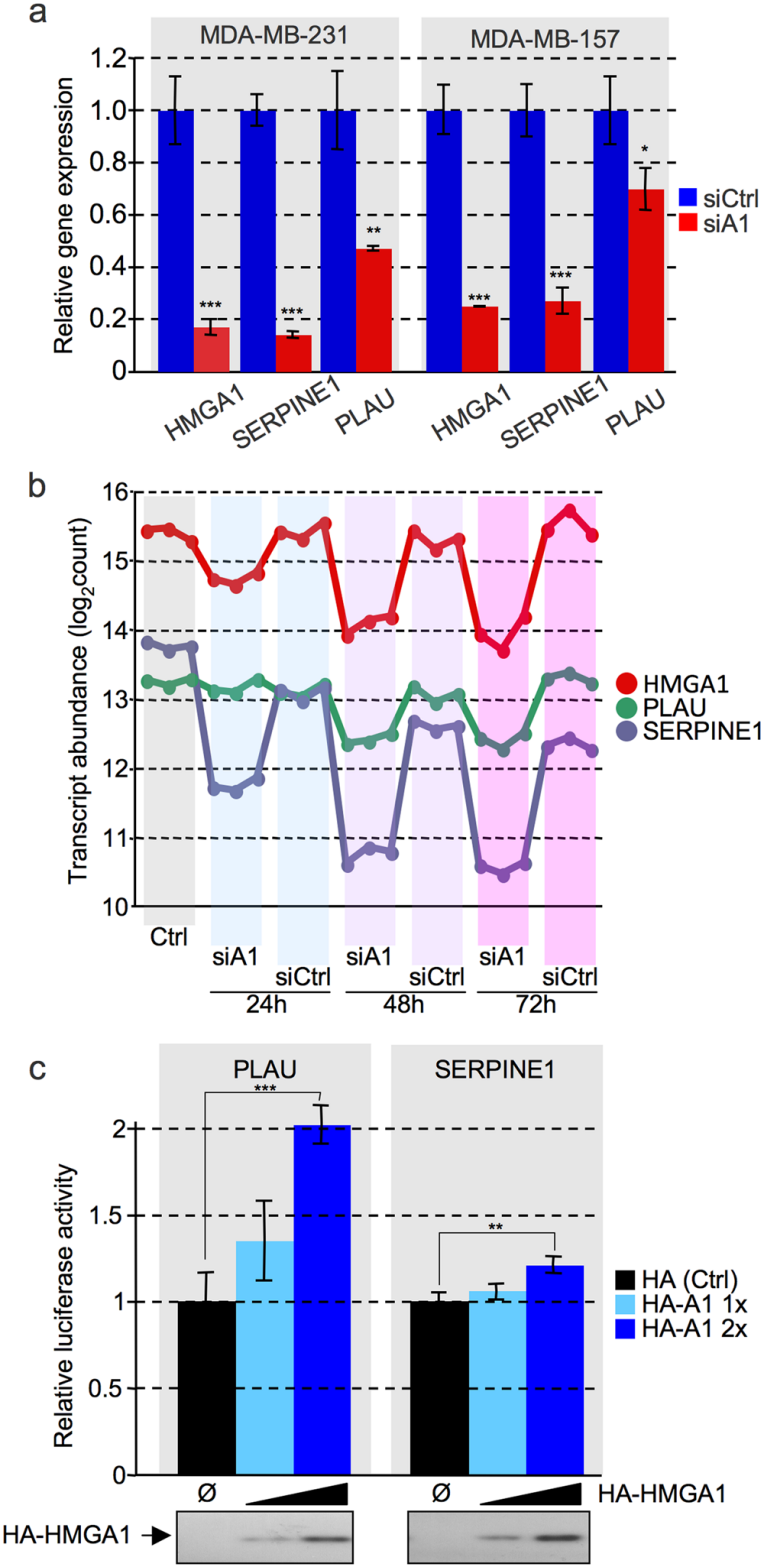
HMGA1 is involved in the transcriptional regulation of PLAU and SERPINE1. (**a**) MDA–MB–231 and MDA–MB–157 cells were treated with siRNA targeting HMGA1 (siA1) or control (siCtrl) siRNA. mRNA expression levels of HMGA1, SERPINE1, and PLAU were analysed by qRT-PCR comparing HMGA1-silenced cells (siA1) versus control cells (siCtrl). Statistical significance was assessed with Student’s t-test (*P < 0.05; **P < 0.01; ***P < 0.001). (**b**) HMGA1, PLAU, and SERPINE1 transcript abundance data were extrapolated from a time course experiments performed with HMGA1 silenced MDA–MB–231 cells analysed by RNA-seq. Ctrl: untreated cells; HMGA1 silenced cells (siA1) and control cells (siCtrl) were treated with siRNA molecules and analysed after 24, 48, or 72 hours. (**c**) Luciferase assays were performed in HEK293T cells transiently cotransfected with 200 ng of the luciferase reporter plasmids containing cis regulatory regions proximal to the transcription start sites of both PLAU and SERPINE1 [pGL4-phPLAU (from −1374 to +29) and pGL4-phSERPINE1 (PAI-1) (from −1410 to +39)] and 0, 200, or 400 ng of the expression plasmid pcDNA3HA-HMGA1a. 10 ng of pRL-CMV *Renilla* luciferase expression vector was included to normalize for transfection efficiencies. Values are reported as relative luciferase activity. Standard deviations are indicated (n = 3). The amounts of transfected HMGA1a in each sample were controlled by Western blot analysis using an anti-HA antibody. Statistical significance was assessed with Student’s t-test (*P < 0.05; **P < 0.01; ***P < 0.001).

It was striking to have found in our secretomic screening the three main members of this system as HMGA1–dependent differentially secreted protein. The three proteins are Plasminogen activator inhibitor 1 (PAI–1, gene name: SERPINE1), Urokinase-type plasminogen activator (uPA, gene name: PLAU), and Urokinase plasminogen activator surface receptor (uPAR, gene name: PLAUR). Our qRT–PCR experiments showed that in MDA–MB–213 shA1_3 cells induced for the silencing of HMGA1, both SERPINE1 and PLAU expression was repressed, while PLAUR seems not to be dependent upon HMGA1 expression levels. The cellular model adopted in this screening is a long–term model, i.e. cells are kept on culture for several days after HMGA1 silencing induction and therefore changes in protein expression levels could be also due to secondary effects, not strictly directly dependent upon HMGA1 expression levels modulation. We therefore evaluated the HMGA1-dependence of PLAU and SERPINE1 by siRNA transfection in MDA–MB–231 cells. As it is possible to observe from Fig. 5a–left side, after 72 h from siRNA treatment, concurrently with a strong silencing of HMGA1 (about 80%), the expression of both PLAU and SERPINE1 is significantly downregulated. The same effect was obtained with another TNBC cell line, i.e. MDA–MB–157 cells (Fig. 5a–right side). To further evaluate a direct link between HMGA1 and PLAU and SERPINE1 expression levels we extracted data obtained from a RNA–seq time course experiment in which HMGA1- silenced MDA-MB-231 cells and their respective controls were collected 24, 48, and 72 hours after siRNA treatment. As can be seen in Fig. 5b, the expression levels of PLAU and SERPINE1 strictly parallel that of HMGA1. Moreover, using a dual luciferase reporter assays performed with promoter sequences of the PLAU and SERPINE1 genes (from −1374 to +29 and from −1410 to +39, respectively) we demonstrated that HMGA1 has a positive modulatory role on these cis regulatory elements (Fig. 5c). Bioinformatic analyses of the 10000 bp upstream the Transcription Start Sites (TSS) of both PLAU and SERPINE1 reveal that there are several long AT–rich DNA regions that could be potential HMGA1 binding sites that could be involved in the modulation of gene expression (Supplementary Figs 3 and 4).

Given that the urokinase plasminogen activator system is involved also in activating intracellular signalling pathways and that one of its main transducers is the Focal adhesion kinase (FAK)^17^ we wondered whether HMGA1 protein could contribute in amplifying this signalling transduction mechanism also by regulating FAK expression. As can be seen from Supplementary Fig. 5, the silencing of HMGA1 in MDA–MB–231 cells leads to a consistent down–regulation of FAK expression levels, thus highlighting a prominent and multifaceted role of HMGA1 in contributing to the urokinase plasminogen activator system signalling.

## Discussion

The main aim of our work was to systematically unravel the HMGA1 impact on breast cancer secretome in order to determine whether HMGA1 could influence cancer aggressiveness by the modulation of secreted proteins. To accomplish this we (i) set up a method for the selective enrichment of secreted protein exploiting a glycoprotein affinity enrichment strategy, (ii) demonstrated that the pool of secreted proteins obtained by HMGA1–expressing cells has a higher capacity in modulating cell motility with respect to those obtained by HMGA1–silenced cells, and (iii) identified those that were differentially secreted by an iTRAQ proteomic differential screening. The vast majority of the proteins that we detected as differentially secreted has a well-defined role in modulating cancer cell motility and aggressiveness. These findings suggest that HMGA1 has a relevant impact in modulating the presence in the extra–cellular environment of key factors involved in cancer aggressiveness. On the basis of these observations we tried to shed light on those factors directly modulated by HMGA1 that could contribute to the acquisition of migratory and invasive properties. Bioinformatic analyses highlighted that the down–regulated proteins were those mostly linked to cancer and cell motility and a Kaplan Meier filtering process helped us to select for proteins clinically linked to a poor prognosis in terms of relapse– and distant metastasis–free survival. Since HMGA1 is an architectural transcriptional factor and its downregulation in TNBC cell lines is responsible for the reversion of the cancer phenotype, we choose to further restrict our study on those proteins transcriptionally linked to HMGA1 in order to focus on early events in the HMGA1–dependent neoplastic cascade. The gene expression of four proteins turned out to be linked to HMGA1 and uPA and PAI–1 drawn our attention.

These proteins belong to the plasminogen activation system. The Urokinase-type plasminogen activator (uPA) is a secreted factor that binds at the outer cell surface its own receptor Urokinase-type plasminogen activator receptor (uPAR)^15–17^. The binding of uPA to uPAR leads to the activation of the former and, after its activation, uPA cleaves plasminogen into plasmin, a serine protease. Plasmin contributes to the extracellular matrix (ECM) remodelling process by directly cleaving several proteins such as laminin and fibronectin and by activating a series of Matrix Metalloprotinases (MMPs)^15–17^. The binding of PAI–1 to uPA inhibits its activity and hence plasmin activation. It has turned clear that the ECM remodelling activity of plasmin is a fundamental process involved in allowing cells to move inside tissues, and hence it is important for cancer cell metastatic spreading. However, the importance of this system in cancer development goes beyond this ECM–linked effect^15–17^. First of all, the ECM degradation causes the release of ECM–entrapped growth factors with a consequent activation of downstream signalling pathways. Moreover, the uPA/uPAR complex is not only involved in plasmin activation but it is also involved in intracellular signalling activation^15–17^. Since uPAR is anchored to the cell surface via a glycosyl phosphatidylinositol (GPI) anchor it lacks a cytosolic domain for the direct transmission of signals and it exploits other mechanisms for the signalling transduction, i.e. it cooperates with integrins, G–protein coupled receptors, and caveolins and lipids rafts. It is of relevance that one of the main pathways activated by uPA/uPAR is the Ras–MAPK/ERK pathway^17^. HMGA1 is both a downstream target and an activator of this pathway^18–21^. Moreover, the MAPK/ERK pathway has been demonstrated to be responsible for the regulation of a key HMGA1 translational regulator, i.e. let–7. Indeed, it can lead to the up regulation of myc that in turn controls the expression of Lin28, an RNA binding protein that is a main “inhibitor” of let–7^22,23^. From these observations is emerging a picture in which HMGA1 is in the centre of an intricate series of self–sustaining feedback loops. The involvement of HMGA1 in the uPA/uPAR functional and signalling network is even deeper. Indeed it is known that HMGA1 transcriptionally regulates MMP–2 and MMP–9, two matrix metalloproteases that are activated by plasmin^24–28^ and that it regulates the expression of caveolin 1 and 2^19^ and focal adhesion kinase (FAK, see Supplementary Fig. 5), which are exploited by uPA/uPAR for intracellular signal transduction.

HMGA1 is a key factor deeply involved in conferring and sustaining cell metastatic behaviours^7^, therefore the possibility of interfering with this HMGA1 circuit could represent a potential tool to counteract cancer metastatic spreading.

## Methods

### Cell Culture

The TNBC cell lines MDA-MB-468, MDA-MB-231, and the MDA-MB-231_shA1_3 clone were grown in DMEM (Dulbecco’s Modified Eagle’s Medium) growth medium containing 10% tetracycline-free FBS (fetal bovine serum), 2 mM L-glutamine, penicillin, and streptomycin. shRNA sequence and the corresponding inducible clone have been previously described^3^. Cells were grown at 37 °C in humidified 5% CO_2_ incubator and collected under sub confluence conditions.

### Collecting conditioned medium

The secretomes were collected as conditioned culture media (CCM). 2.7 million cells (NI and I MDA-MB-231 shA1_3 cells; NI: not induced for the silencing of HMGA1; I: induced for the silencing of HMGA1) were seeded in 175 cm^2^ culture flasks (*Corning*) and left three days in normal growth medium (10 × 175 cm^2^ cell culture flask for each replica – biological triplicate). After 72 h cells were washed twice (each 3 h) with serum free media and then left 24 h before CCM collection. CCM were centrifuged at 1880g for 10 min at 4 °C and the supernatant were filtered through a 0.22 μm filter device and stored at −80 °C. CCM were concentrated to about 1.5 mL on Amicon Ultra-15, 10000 MWCO centrifugal filter units (*Millipore*) and in parallel the buffer was changed to lectin binding buffer (20 mM Tris, 0.15 M NaCl, 1 mM MnCl_2_, and 1 mM CaCl_2_, pH 7.4).

### Lectin affinity capture of glycoproteins

500 μL agarose bound Con A and 500 μL agarose bound WGA (*Vector Laboratories, cat. AL1003 and AL1023*) were mixed and equilibrated with lectin binding buffer. 500 μg CCM proteins were incubated with agarose bound lectins overnight at 4 °C in lectin binding buffer. The day after, unbound proteins were washed out with 1 mL lectin binding buffer for three times. Bound proteins were eluted with 1 mL elution buffer (20 mM Tris, 0.5 M NaCl, 0.4 M methyl-α-D-mannopyroside, and 0.5 M N-acetyl-glucosamine, pH 7.0) twice. Buffer was changed to 10 mM Tris, 10 mM NaCl, pH 7.0 by ultrafiltration. Protein concentrations were determined by the Lowry assay (*Pierce*, *Thermo Scientific)*.

### Viability assay

Proliferation of MDA-MB-231 cells and the NI and I shA1_3 clones during serum starvation was monitored using the MTS assay. 5000 cells were seeded into 96-well plate and left grown for 72 h. Cell viability was determined by CellTiter 96 Aqueous One Solution Assay (*Promega*) according to manufacturer instructions. Briefly, after 0, 24, 48, 72, or 96 h of incubation in the growth medium with or without serum, 120 μL of MTS solution (MTS reagent diluted 1:6 in PBS containing glucose 4,5 g/L) was added into each well, the plate was incubated for 2.5 h at 37 °C in 5% CO_2_ atmosphere, and then absorbance at 490 nm was recorded with a 96-well plate ELISA reader. The same cell viability assay was performed on MDA-MB-468 cells treated with enriched glycoproteins (0.35 µg/well) obtained from MDA-MB-231_shA1_3 expressing or not expressing HMGA1 (NI and I, respectively). Cells were treated after 24 h from seeding.

### Migration Assay

Wound healing and transwell assays were performed as previously described^3^. MDA-MB-468 cells were cultured to sub-confluence on 9.6 cm^2^ wells. After 48 h from seeding, culture medium was replaced either with FBS containing medium or without it (serum starvation) and cells were treated with glycoproteins from NI and I cells (3.5 µg/well). After 24 h, the cells were scraped with a 200 μl tip and wound closure was followed for 24 h. For transwell migration assay, 24-well PET inserts were used (8.0 μm pore size, *Falcon*) and 40000 cells (MDA-MB-468) were resuspended in upper transwell chambers in 0.5% FBS containing medium and allowed to migrate towards a serum gradient (10%), additioned with glycoproteins (1.31 µg) from MDA–MB–231 shA1_3 NI and I cells in the lower chamber for 24 h. Values reported are the averages of experiment performed in quadruplicates. Controls were performed using the buffer in which glycoproteins were conditioned (10 mM Tris, 10 mM NaCl, pH 7.0).

### Protein Extraction and Western Blot Analysis

Cells were washed in chilled PBS and lysed directly in the dish using SDS sample buffer. Western Blot analyses were performed according to standard procedures. Immunoblotting were performed using antibody to HMGA1 developed in our laboratories^5^ at a 1:500 dilution, anti-HA antibody (*Sigma*, cat. N° H9658) at a 1:1000 dilution, and HRP-conjugated anti-rabbit –mouse secondary antibodies at a 1:5000 dilution (*Sigma*, cat. N° A0545 and N° A9044, respectively). Detection was performed using chemilumiscence ECL reagent (*Pierce, Thermo Scientific*).

### 1D gel electrophoresis and in-gel digestion

Glycoproteins (50 μg each lane) were subjected to SDS-PAGE (T = 15%), the run was stopped just after proteins entered the running gel and stained with Colloidal Coomassie Blue (10% (w/v) phosphoric acid, 10% (w/v) ammonium sulphate, 20% (v/v) anhydrous methanol and 0.12% (w/v) Coomassie Brilliant Blue G-250). After extensive destaining with water, each lane was excised and cut into 1 mm cubes and destained by incubation in a 50% acetonitrile solution. Gel pieces were dehydrated with two 10 min washes with acetonitrile and dried. The dried gel pieces were swollen in 10 mM dithiothreitol (DTT), 100 mM triethylammonium bicarbonate (TEAB), left 1 h at 60 °C; and then treated with 50 mM iodoacetamide (IAA) in 100 mM TEAB for 30 min in the dark and at RT. Gel pieces were sequentially washed at RT in 100 mM TEAB (15 min), in 20 mM TEAB in 50% Acetonitrile (15 min), in Acetonitrile (5 min), and finally dried. Gel pieces were then swollen in 20 μL of 50 mM TEAB containing 0.05 μg/μL trypsin (*Trypsin gold, Mass Spectrometry grade, Promega*). 50 mM TEAB was added to cover the gel pieces, and they were incubated o/n at 37 °C. Peptides were extracted with a 50% Acetonitrile, 0.1% (v/v) formic acid solution (15 min) for three times in a bath sonicator and then dried.

### iTRAQ labelling

Each purified glycoprotein sample was dissolved with 500 μl of 0.1% formic acid, desalted on SepPak C18 Cartridges (Waters) and dried under vacuum.

Each sample was suspended in 70 μl of dissolution buffer (iTRAQ Reagent Multi-Plex Kit, AB Sciex) and divided in two aliquots, each containing 25 μg of peptides. iTRAQ labelling was carried out essentially as previously described^29^ with a tag swapping strategy: Replicate 1 – I:114,116; NI:115,117/Replicate 2 – I:115,117; NI:116,114; Replicate 3 – I:116,114; NI:117,115. 1 µg of every tagged sample was analysed by LC-MS/MS and intensities of the base peak chromatograms (BPC) were used to verify peptide quantification. The four samples from the same biological replicate were pooled with a 1:1:1:1 ratio and dried under vacuum.

### SCX fractionation

SCX fractionation was carried out as previously described^29^. The stepwise elution was carried out with: 50, 80, 110, 140, 170, 200, 250, and 350 mM KCl. SCX fractions were desalted (see above) and dried under vacuum.

### Mass spectrometry analysis

LC-MS/MS analyses were performed with a LTQ-Orbitrap XL mass spectrometer as previously described^29^.

SCX fractions were dissolved in 40 μl of 3% acetonitrile/0.1% formic acid and loaded into homemade uncoated pico-frit column (New Objective) packed with C18 material (Aeris Peptide 3.6 μm XB-C18, Phenomenex): 4 μl for fractions eluted with 50, 80, and 110 mM KCl, 6 μl for those eluted with 140 and 170 mM KCl, 8 μl for the fractions eluted with 200, 250, and 350 mM KCl.

The instrument was operating in a data-dependent mode, with a Top 3 CID/HCD method^30^. All identified peptides were used to create a static exclusion list that was then inserted into the instrument method for a second analysis.

### Data analysis

All files were analysed in a MudPIT protocol with Proteome Discoverer 1.4 software (Thermo Fisher Scientific) interfaced to Mascot (version 2.2.4, Matrix Science, London, UK) and SequestHT (Thermo Fisher Scientific). The searches were performed against the Uniprot Human protein database (version 2013.11.13, 88473 sequences) and the results obtained from the two search engines were combined into a single output msf file.

Enzyme specificity: Trypsin, up to 1 missed cleavage. Precursor and fragment mass tolerances: 10 ppm and 0.6 Da, respectively. Variable modification: oxidation of methionine. Fixed modifications: carbamidomethyl cysteine and iTRAQ 4plex labelling on N-terminus and lysine residues. Percolator was used to assess the confidence of identification. Proteins were considered as correctly identified if at least 2 peptides/protein were identified with individual q values ≤ 0.05 and grouped into families according to the principle of maximum parsimony. Quantification of peptides was performed by Proteome Discoverer using the intensities of the reporter ions, while protein quantification was obtained as the median value of all quantified peptides for a given protein.

### Quantitative Real-Time PCR (qRT-PCR)

qRT-PCR analyses were performed essentially as previously described^3^. Primers sequences are reported in supplementary information.

### Functional analysis

Functional analysis has been performed using Ingenuity Pathway Analysis (Ingenuity® Systems, www.ingenuity.com) and David^31,32^. Results with corrected pValue ≤ 0.05 were considered significant. For the enrichment of secreted and glycosylated proteins, starting from David Ease results we selected the enriched terms related to the processes of interest. The selected terms are: -“glycoprotein”, “glycosylation site”, “signal”, “extracellular exosome”, “extracellular space” and “secreted”.

### Luciferase assay

HEK-293T cells were plated at density of 350.000 cells per 35-mm-diameter culture dish and processed 42 h after standard calcium phosphate transfection. Cells were transfected with 200 ng of the reporter constructs and 200 or 400 ng of pcDNA3HA-HMGA1a vector. In order to normalize for transfection efficiencies, 10 ng of pRL-CMV Renilla luciferase expression vector (Promega) were transfected into the cells. The reporter constructs were the following: pGL4-phPLAU (From −1374 to +29 relative to the predominant transcription start site) and pGL4-phSERPINE1 (PAI-1) (From −1410 to +39) (DNA Bank RIKEN BioResource Center). The assays were performed with dual-luciferase reporter assay system (Promega) according to the manufacturer’s instruction. Western blot analyses were performed on samples normalized for transfection efficiencies.

## Electronic supplementary material

Supplementary info

Supplementary info

Supplementary info

Supplementary info
