# Supplementary material for: HMGA1 regulates the Plasminogen activation system in the secretome of breast cancer cells
Source: Sci Rep. 2017 Sep 18;7:11768. doi: 10.1038/s41598-017-11409-4 (PMC5603555; doi:10.1038/s41598-017-11409-4)
Supplement: Supplementary file 1 — Supplementary info [file 41598_2017_11409_MOESM1_ESM.pdf]

HMGA1 regulates the Plasminogen activation system in the secretome of breast cancer cells

Giulia Resmini[1,§,#], Serena Rizzo[1,#], Cinzia Franchin[2,3], Rossella Zanin[1], Carlotta Penzo[1], Silvia Pegoraro[1], Yari Ciani[4], Silvano Piazza[4,£], Giorgio Arrigoni[2,3], Riccardo Sgarra[1,*] and Guidalberto Manfioletti[1,*]

[1]Department of Life Sciences, University of Trieste, Trieste, Italy

[2]Department of Biomedical Sciences, University of Padova, Padova, Italy.

[3]Proteomics Center, University of Padova and Azienda Ospedaliera di Padova, Padova, Italy.

[4]National Laboratory CIB (LNCIB), Area Science Park, Trieste, Italy

[§]Present address: I.R.C.C.S. Policlinico San Donato, Milano, Italy

[£]Present address: Bioinformatics Core Facility, Centre for Integrative Biology, CIBIO, University of Trento, Trento, Italy

[#]these authors contributed equally.

[*]co–last and co-corresponding authors.

Correspondence:

Guidalberto Manfioletti & Riccardo Sgarra

Department of Life Sciences, University of Trieste

Via L. Giorgieri, 5 - 34127 Trieste

Italy

Phone: +39 040 558-8720 / -8721

E-mail: rsgarra@units.it / manfiole@units.it

Supplementary Figure legends

Supplementary Figure S1. Effect of serum starvation on the growth of MDA-MB-231 and MDA–MB–231 shA1_3 cells and the evaluation by SDS–PAGE of a protocol for the enrichment of glycosylated proteins. a) Metabolic activity of MDA-MB-231 and MDA-MB-231_shA1_3 not induced (NI) and induced (I – 10 days). Cells were seeded at a density of 5000 cells per well in 96-multiwell. After 72 hours, complete medium has been removed and replaced with serum-free medium for the serum starvation (SS). As a control, cells were also maintained in complete medium. MTS assay was performed at 0, 24, 48, 72, and 96 h after serum starvation. Values shown are averages +/– SD (n=3). The metabolic activity of cells at time 0 was set to 100% arbitrarily. The dashed vertical line indicates the 30h time chosen for the collection of secreted proteins. b) Silver staining of proteins separated by SDS-PAGE (T=15%) to verify glycoproteins purification protocol. CM Input: protein concentrate obtained from the culture medium (CM). Samples purified with a control resin (Empty Resin, i.e. without lectins) or with a mixture of ConA– and WGA–derivatized resins are shown (ConA+WGA). Unbound: unbound proteins (flow through), lanes 2 and 5. Competitive elution: proteins retained by the resin and eluted by competitive elution with methyl-α-D-mannopyroside and N-acetyl-glucosamine, lanes 3 and 6. SDS elution: protein retained by the resin and eluted by SDS, lanes 4 and 7. Molecular weights markers (kDa) are shown on the left. c) The conA+WGA affinity purified proteins (lane 6, panel b) have been identified by LC–MS/MS analyses and subjected to bioinformatic evaluation (gene-annotation enrichment analysis).

Supplementary Figure S2. Checking (i) the efficiency of HMGA1 silencing in MDA-MB-231_shA1_3 cells, (ii) the normalization of protein samples subjected to iTRAQ screening, and (iii) the enrichment of secreted/glycosylated proteins in the ConA+WGA affinity purification. a) 12 out of 60 independent 175 cm^2 cell culture flask were randomly selected for WB analyses to check for HMGA1 expression levels in MDA–MB–231 shA1_3 I and NI cells. After collection of culture media, total protein lysates obtained from cells were analyzed by SDS PAGE (T=15%) and western blot. A blue coomassie (BC) stained gel is shown to check protein quantity normalization. The expression of HMGA1 was detected by anti-HMGA1 antibody (α–A1). Molecular weight markers (kDa) are shown on the right. b) Blue Coomassie staining of proteins separated by SDS-PAGE (T=15%) to verify glycoproteins quantification and normalization by the

Lowry method. Equivalent amounts (4.5 μg) of the glycoproteins purified in the three biological replicates (NI and I) were loaded. c) The conA+WGA affinity purified proteins have been identified by the iTRAQ LC–MS/MS screening. All the identified proteins have been subjected to bioinformatic evaluation (gene-annotation enrichment analysis).

Supplementary Figure S3. Transcription factor motif analysis of the PLAU promoter/enhancer region (about 10 kbp upstream the transcription start site). The analysis of the promoters sequences was performed through Biobase Transfac using the Match analysis method. Promoter sequences were obtained from the Transfac internal database (version 2016.3) selecting a distance of 10kb upstream and 1kb downstream the TSS. The sequence of the *PLAU* promoter/enhancer region is shown. Putative transcription factor binding sites are evidenced with arrows indicating the binding orientation.

Supplementary Figure S4. Transcription factor motif analysis of the SERPINE1 promoter/enhancer region (about 10 kbp upstream the transcription start site). The analysis of the promoters sequences was performed through Biobase Transfac using the Match analysis method. Promoter sequences were obtained from the Transfac internal database (version 2016.3) selecting a distance of 10kb upstream and 1kb downstream the TSS.The sequence of the *SERPINE1* promoter/enhancer region is shown. Putative transcription factor binding sites are evidenced with arrows indicating the binding orientation.

Supplementary figure S5. Focal Adhesion Kinase (FAK) expression levels are linked to HMGA1. MDA–MB–231 cells have been treated with siRNA targeting HMGA1 (siA1) or with control siRNA (siCtrl). Proteins have been extracted with SDS lysis buffer and analysed by SDS–PAGE and western blot. FAK has been detected using a α-FAK antibody. The membrane after western blot has been stained with red ponceau and it is show as a loading control. Molecular markers (kDa) are shown on the right.

Supplementary Figure 1

a

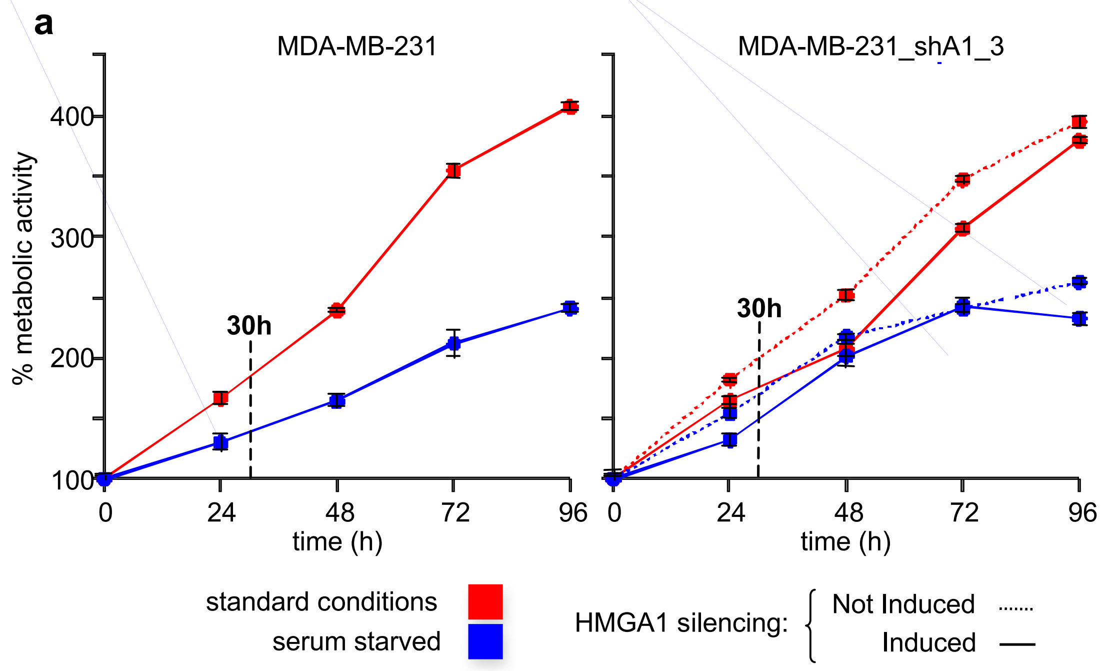

b

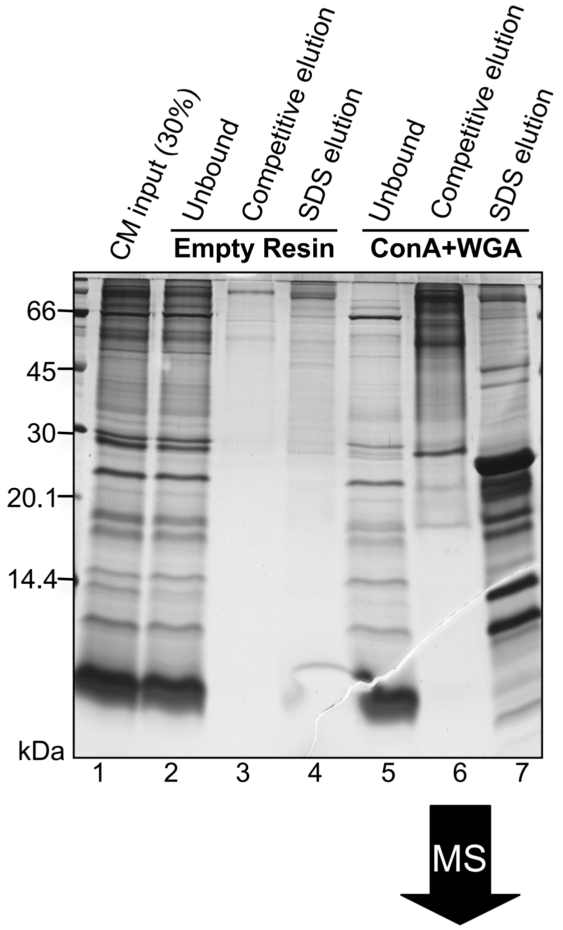

c

Term	Count	%	PValue	Fold Enrichment	FDR
Glycoprotein	186	85,71	7,06E-89	3,87	9,22E-86
glycosylation site:N-linked (GlcNAc...)	179	82,49	3,18E-84	3,93	5,04E-81
Signal	174	80,18	1,09E-80	3,98	1,43E-77
extracellular exosome	150	69,12	3,96E-71	4,50	5,25E-68
extracellular space	108	49,77	1,29E-62	6,66	1,71E-59
Secreted	94	43,32	1,36E-39	4,65	1,78E-36

Supplementary Figure 2

a

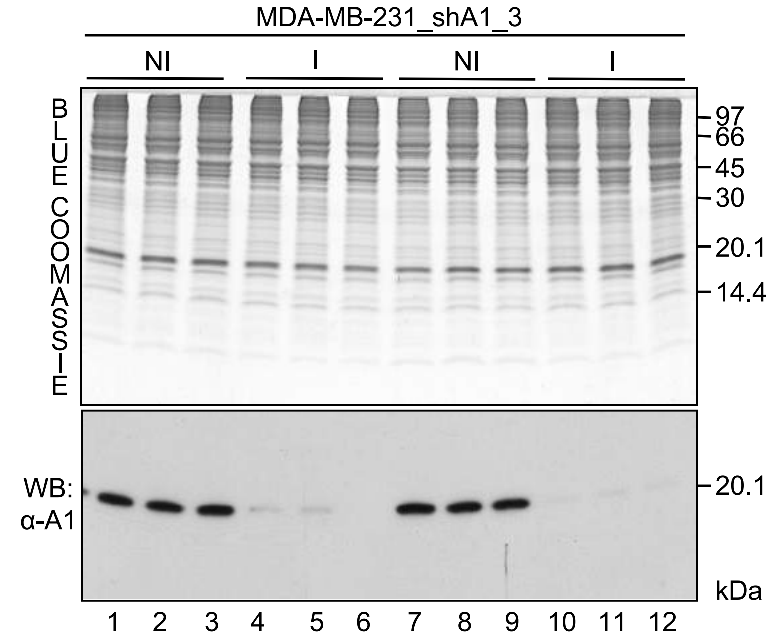

b

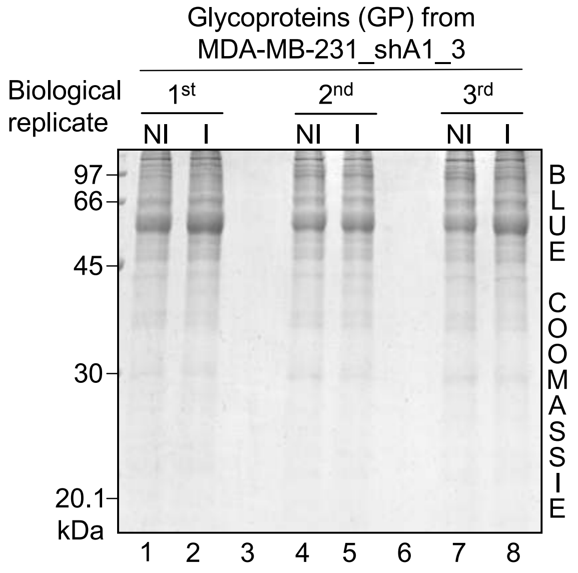

c

iTRAQ data: identified proteins

Term	Count	%	PValue	Fold Enrichm	FDR
extracellular exosome	155	71,76	4,60E-77	4,652226	6,13E-74
Signal	153	70,83	5,64E-58	3,504377	7,43E-55
Glycoprotein	158	73,15	1,99E-57	3,3079807	2,63E-54
glycosylation site:N-linked (GlcNAc...)	149	68,98	1,30E-51	3,2687186	2,08E-48
extracellular space	96	44,44	1,76E-50	6,0130331	2,35E-47
Secreted	88	40,74	7,71E-34	4,2671002	1,02E-30

Supplementary Figure S3
PLAU Promoter/Enhancer analysis

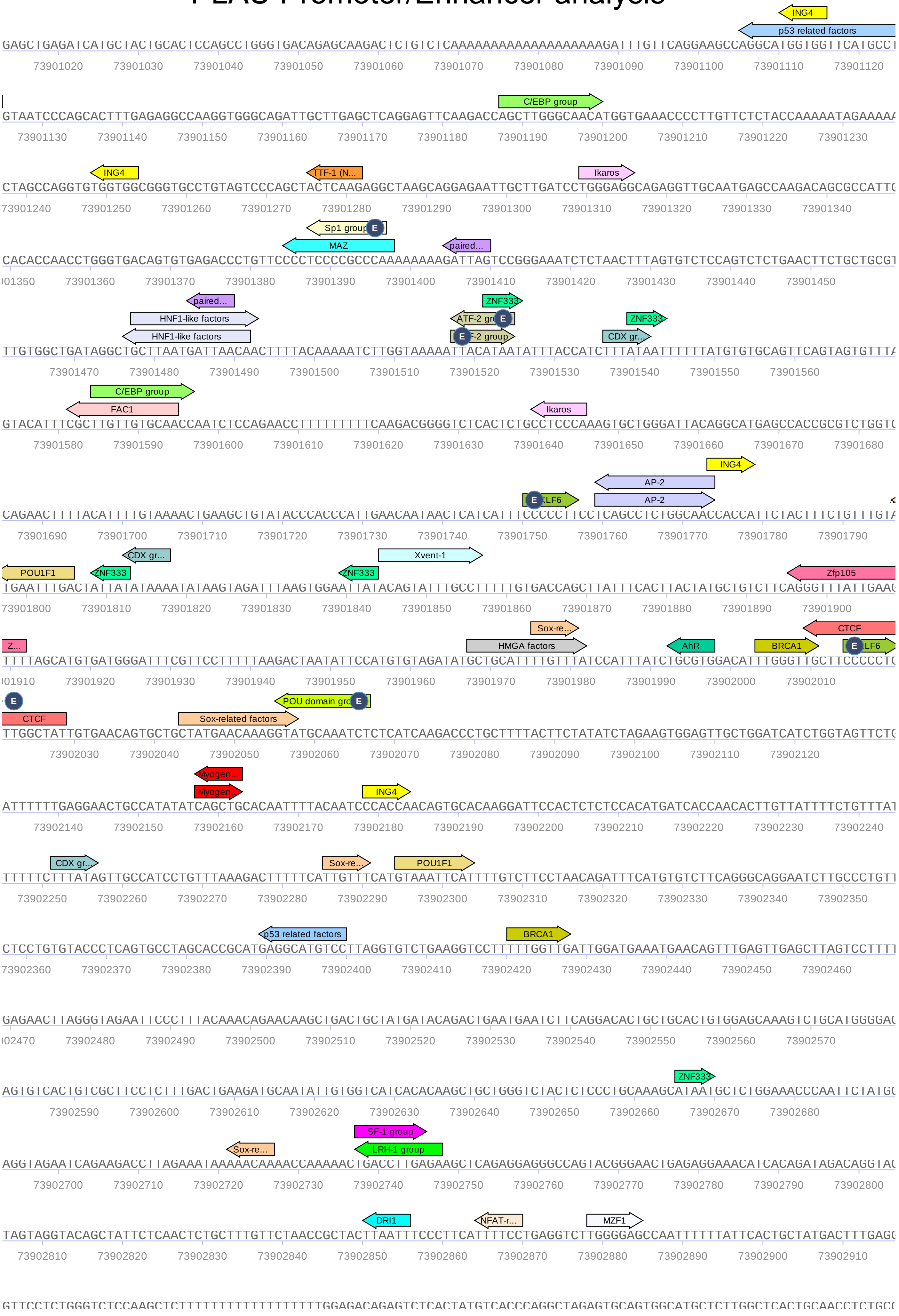

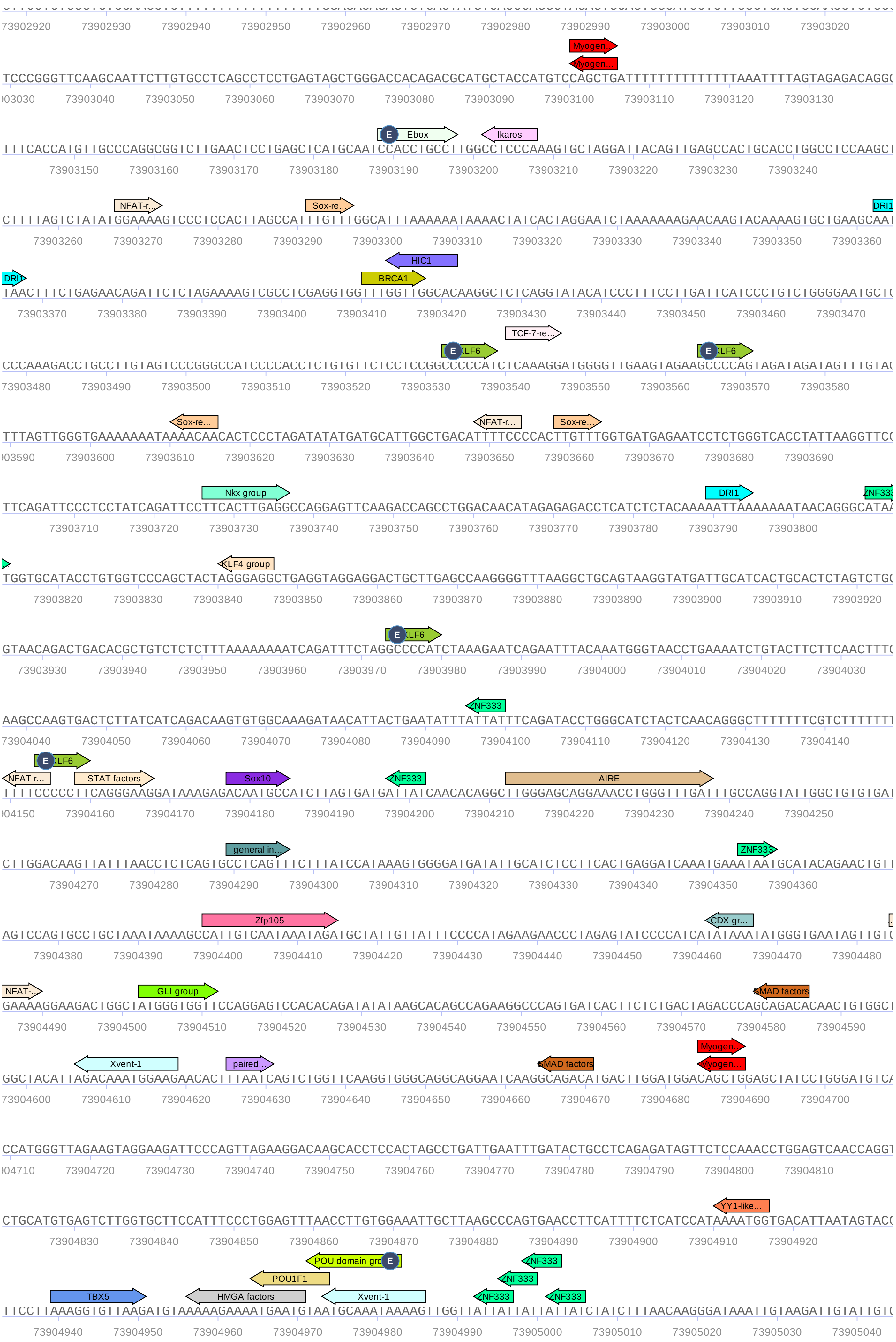
73902920 73902930 73902940 73902950 73902960 73902970 73902980 73902990 73903000 73903010 73903020
Myogen...
Myogen...
TCCCGGGTTCAAGCAATTCTTGTGCCTCAGCCTCCTGAGTAGCTGGGACCACAGACGCATGCTACCATGTCCAGCTGATTTTTTTTTTTTTTAAATTTTAGTAGAGACAGGC
03030 73903040 73903050 73903060 73903070 73903080 73903090 73903100 73903110 73903120 73903130
E Ebox
Ikaros
TTTCACCATGTTGCCCAGGCGGTCTTGAACTCCTGAGCTCATGCAATCCACCTGCCTTGGCCTCCCAAAGTGCTAGGATTACAGTTGAGCCACTGCACCTGGCCTCCAAGCT
73903150 73903160 73903170 73903180 73903190 73903200 73903210 73903220 73903230 73903240
NFAT-r...
Sox-re...
DRI1
CTTTTAGTCTATATGGAAAAGTCCCTCCACTTAGCCATTTGTTTGGCATTTAAAAAATAAAACTATCACTAGGAATCTAAAAAAAGAACAAGTACAAAAGTGCTGAAGCAAT
73903260 73903270 73903280 73903290 73903300 73903310 73903320 73903330 73903340 73903350 73903360
DRI1
HIC1
BRCA1
TAACTTTCTGAGAACAGATTCTCTAGAAAAGTCGCCTCGAGGTGGTTTGGTTGGCACAAGGCTCTCAGGTATACATCCCTTTCCTTGATTCATCCCTGTCTGGGGAATGCTG
73903370 73903380 73903390 73903400 73903410 73903420 73903430 73903440 73903450 73903460 73903470
TCF-7-re...
E KLF6
E KLF6
CCCAAAGACCTGCCTTGTAGTCCCGGGCCATCCCCACCTCTGTGTTCTCCTCCGGCCCCCATCTCAAAGGATGGGGTTGAAGTAGAAGCCCCAGTAGATAGATAGTTTGTAC
73903480 73903490 73903500 73903510 73903520 73903530 73903540 73903550 73903560 73903570 73903580
Sox-re...
NFAT-r...
Sox-re...
TTTAGTTGGGTGAAAAAAATAAAACAACACTCCCTAGATATATGATGCATTGGCTGACATTTTCCCCACTTGTTTGGTGATGAGAATCCTCTGGGTCACCTATTAAGGTTCC
03590 73903600 73903610 73903620 73903630 73903640 73903650 73903660 73903670 73903680 73903690
Nkx group
DRI1
ZNF333
TTCAGATTCCCTCCTATCAGATTCCTTCACTTGAGGCCAGGAGTTCAAGACCAGCCTGGACAACATAGAGAGACCTCATCTCTACAAAAATTAAAAAAATAACAGGGCATAA
73903710 73903720 73903730 73903740 73903750 73903760 73903770 73903780 73903790 73903800
KLF4 group
TGGTGCATACCTGTGGTCCCAGCTACTAGGGAGGCTGAGGTAGGAGGACTGCTTGAGCCAAGGGGTTTAAGGCTGCAGTAAGGTATGATTGCATCACTGCACTCTAGTCTGG
73903820 73903830 73903840 73903850 73903860 73903870 73903880 73903890 73903900 73903910 73903920
E KLF6
GTAACAGACTGACACGCTGTCTCTCTTTAAAAAAAATCAGATTTCTAGGCCCCATCTAAAGAATCAGAATTTACAAATGGGTAACCTGAAAATCTGTACTTCTTCAACTTTC
73903930 73903940 73903950 73903960 73903970 73903980 73903990 73904000 73904010 73904020 73904030
ZNF333
AAGCCAAGTGACTCTTATCATCAGACAAGTGTGGCAAAGATAACATTACTGAATATTTATTATTTCAGATACCTGGGCATCTACTCAACAGGGCTTTTTTTCGTCTTTTTTT
73904040 73904050 73904060 73904070 73904080 73904090 73904100 73904110 73904120 73904130 73904140
E KLF6
NFAT-r...
STAT factors
Sox10
ZNF333
AIRE
TTTTCCCCCTTCAGGGAAGGATAAAGAGACAATGCCATCTTAGTGATGATTATCAACACAGGCTTGGGAGCAGGAAACCTGGGTTTGATTTGCCAGGTATTGGCTGTGTGAT
04150 73904160 73904170 73904180 73904190 73904200 73904210 73904220 73904230 73904240 73904250
general in...
ZNF333
CTTGGACAAGTTATTTAACCTCTCAGTGCCTCAGTTTCTTTATCCATAAAGTGGGGATGATATTGCATCTCCTTCACTGAGGATCAAATGAAATAATGCATACAGAACTGTT
73904270 73904280 73904290 73904300 73904310 73904320 73904330 73904340 73904350 73904360
Zfp105
CDX gr...
AGTCCAGTGCCTGCTAAATAAAAGCCATTGTCAATAAATAGATGCTATTGTTATTTCCCCATAGAAGAACCCTAGAGTATCCCCATCATATAAATATGGGTGAATAGTTGTG
73904380 73904390 73904400 73904410 73904420 73904430 73904440 73904450 73904460 73904470 73904480
NFAT-...
GLI group
SMAD factors
GAAAAGGAAGACTGGCTATGGGTGGTTCCAGGAGTCCACACAGATATATAAGCACAGCCAGAAGGCCCAGTGATCACTTCTCTGACTAGACCCAGCAGACACAACTGTGGCT
73904490 73904500 73904510 73904520 73904530 73904540 73904550 73904560 73904570 73904580 73904590
Myogen...
Xvent-1
paired...
SMAD factors
Myogen...
GGCTACATTAGACAAATGGAAGAACACTTTAATCAGTCTGGTTCAAGGTGGGCAGGCAGGAATCAAGGCAGACATGACTTGGATGCAGCTGGAGCTATCCTGGGATGTCA
73904600 73904610 73904620 73904630 73904640 73904650 73904660 73904670 73904680 73904690 73904700
CCATGGGTTAGAAGTAGGAAGATTCCCAGTTAGAAGGACAAGCACCTCCACTAGCCTGATTGAATTTGATACTGCCTCAGAGATAGTTCTCCAAACCTGGAGTCAACCAGGT
04710 73904720 73904730 73904740 73904750 73904760 73904770 73904780 73904790 73904800 73904810
YY1-like...
CTGCATGTGAGTCTTGGTGCTTCCATTTCCCTGGAGTTTAACCTTGTGGAAATTGCTTAAGCCCAGTGAACCTTCATTTTCTCATCCATAAAATGGTGACATTAATAGTACC
73904830 73904840 73904850 73904860 73904870 73904880 73904890 73904900 73904910 73904920
POU domain gro E
ZNF333
POU1F1
ZNF333
TBX5
HMGA factors
Xvent-1
ZNF333
ZNF333
TTCCTTAAAGGTGTTAAGATGTAAAAAGAAAATGAATGTAATGCAAATAAAAGTTGGTTATTATTATTATTATCTATCTTTAACAAGGGATAAATTGTAAGATTGTATTGTC
73904940 73904950 73904960 73904970 73904980 73904990 73905000 73905010 73905020 73905030 73905040

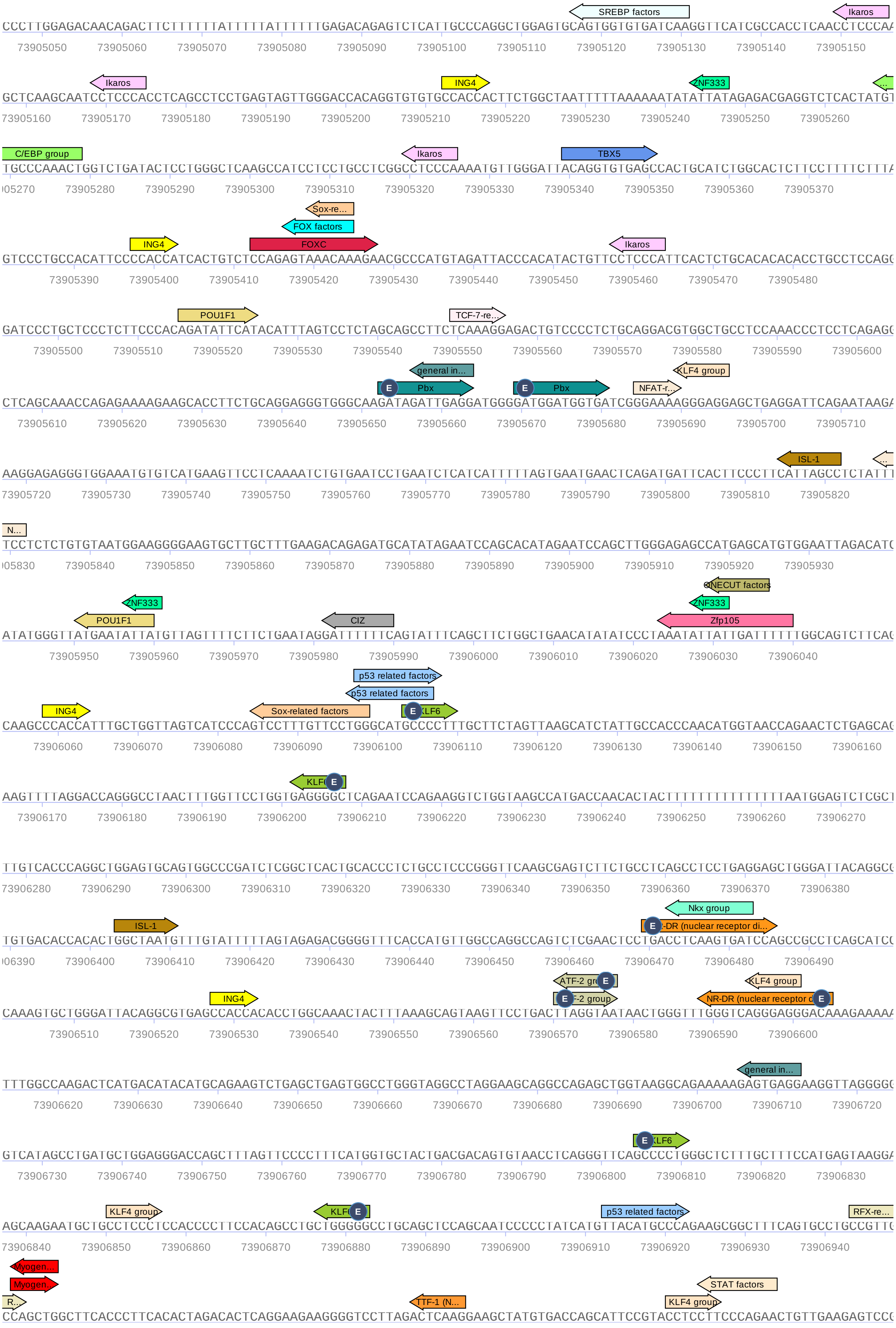

SREBP factors
Ikaros
ING4
ZNF333
C/EBP group
TBX5
Sox-re...
FOX factors
FOXC
POU1F1
TCF-7-re...
general in...
Pbx
KLF4 group
NFAT-r...
ISL-1
N...
ONECUT factors
Zfp105
CIZ
p53 related factors
Sox-related factors
KLF6
Nkx group
NR-DR (nuclear receptor di...
ATF-2 group
NR-DR (nuclear receptor d
RFX-re...
Myogen...
R...
TTF-1 (N...
STAT factors

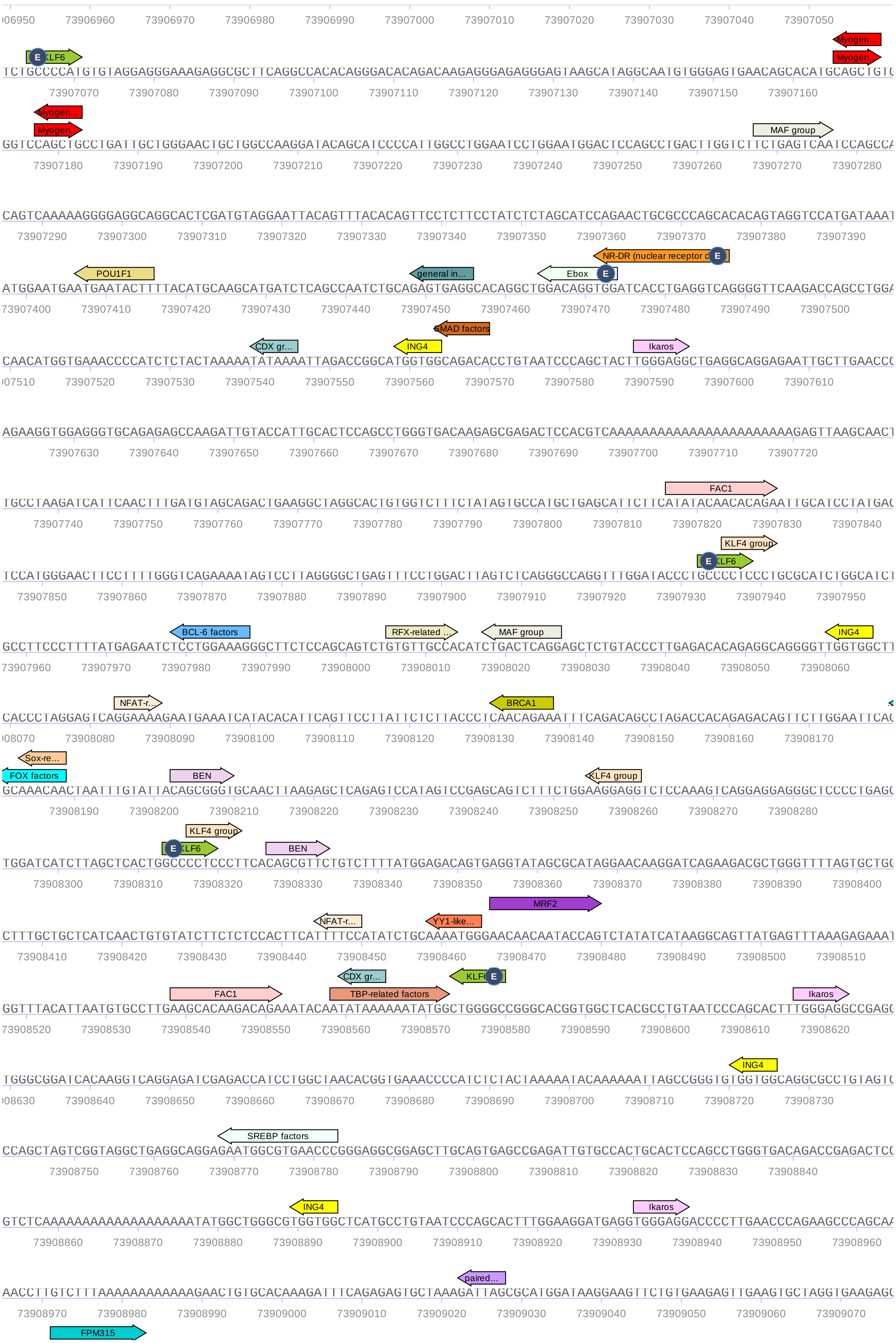

)06950 73906960 73906970 73906980 73906990 73907000 73907010 73907020 73907030 73907040 73907050
KLF6
Myogen...
Myogen...
TCTGCCCCATGTGTAGGAGGGAAAGAGGCGCTTCAGGCCACACAGGGACACAGACAAGAGGGAGAGGGAGTAAGCATAGGCAATGTGGGAGTGAACAGCACATGCAGCTGTC
73907070 73907080 73907090 73907100 73907110 73907120 73907130 73907140 73907150 73907160
Myogen...
Myogen...
MAF group
GGTCCAGCTGCCTGATTGCTGGGAACTGCTGGCCAAGGATACAGCATCCCCATTGGCCTGGAATCCTGGAATGGACTCCAGCCTGACTTGGTCTTCTGAGTCAATCCAGCCA
73907180 73907190 73907200 73907210 73907220 73907230 73907240 73907250 73907260 73907270 73907280
CAGTCAAAAAGGGGAGGCAGGCACTCGATGTAGGAATTACAGTTTACACAGTTCCTCTTCCTATCTCTAGCATCCAGAACTGCGCCCAGCACACAGTAGGTCCATGATAAAT
73907290 73907300 73907310 73907320 73907330 73907340 73907350 73907360 73907370 73907380 73907390
NR-DR (nuclear receptor d
POU1F1
general in...
Ebox
ATGGAATGAATGAATACTTTTACATGCAAGCATGATCTCAGCCAATCTGCAGAGTGAGGCACAGGCTGGACAGGTGGATCACCTGAGGTCAGGGGTTCAAGACCAGCCTGGA
73907400 73907410 73907420 73907430 73907440 73907450 73907460 73907470 73907480 73907490 73907500
SMAD factors
CDX gr...
ING4
Ikaros
CAACATGGTGAAACCCCATCTCTACTAAAAATATAAAATTAGACCGGCATGGTGGCAGACACCTGTAATCCCAGCTACTTGGGAGGCTGAGGCAGGAGAATTGCTTGAACCC
)07510 73907520 73907530 73907540 73907550 73907560 73907570 73907580 73907590 73907600 73907610
AGAAGGTGGAGGGTGCAGAGAGCCAAGATTGTACCATTGCACTCCAGCCTGGGTGACAAGAGCGAGACTCCACGTCAAAAAAAAAAAAAAAAAAAAAAAAGAGTTAAGCAACT
73907630 73907640 73907650 73907660 73907670 73907680 73907690 73907700 73907710 73907720
FAC1
TGCCTAAGATCATTCAACTTTGATGTAGCAGACTGAAGGCTAGGCACTGTGGTCTTTCTATAGTGCCATGCTGAGCATTCTTCATATACAACACAGAATTGCATCCTATGAC
73907740 73907750 73907760 73907770 73907780 73907790 73907800 73907810 73907820 73907830 73907840
KLF4 group
KLF6
TCCATGGGAACTTCCTTTTGGGTCAGAAAATAGTCCTTAGGGGCTGAGTTTCCTGGACTTAGTCTCAGGGCCAGGTTTGGATACCCTGCCCCTCCCTGCGCATCTGGCATCT
73907850 73907860 73907870 73907880 73907890 73907900 73907910 73907920 73907930 73907940 73907950
BCL-6 factors
RFX-related ...
MAF group
ING4
GCCTTCCCTTTTTATGAGAATCTCCTGGAAAGGGCTTCTCCAGCAGTCTGTGTTGCCACATCTGACTCAGGAGCTCTGTACCCTTGAGACACAGAGGCAGGGGTTGGTGGCTT
73907960 73907970 73907980 73907990 73908000 73908010 73908020 73908030 73908040 73908050 73908060
NFAT-r...
BRCA1
CACCCTAGGAGTCAGGAAAAGAATGAAATCATACACATTCAGTTCCTTATTCTCTTACCCTCAACAGAAATTTCAGACAGCCTAGACCACAGAGACAGTTCTTGGAATTCAC
)08070 73908080 73908090 73908100 73908110 73908120 73908130 73908140 73908150 73908160 73908170
Sox-re...
FOX factors
BEN
KLF4 group
GCAAACAACTAATTTGTATTACAGCGGGTGCAACTTAAGAGCTCAGAGTCCATAGTCCGAGCAGTCTTTCTGGAAGGAGGTCTCCAAAGTCAGGAGGAGGGCTCCCCTGAGC
73908190 73908200 73908210 73908220 73908230 73908240 73908250 73908260 73908270 73908280
KLF4 group
KLF6
BEN
TGGATCATCTTAGCTCACTGGCCCCTCCCTTCACAGCGTTCTGTCTTTTATGGAGACAGTGAGGTATAGCGCATAGGAACAAGGATCAGAAGACGCTGGGTTTTAGTGCTGC
73908300 73908310 73908320 73908330 73908340 73908350 73908360 73908370 73908380 73908390 73908400
MRF2
NFAT-r...
YY1-like...
CTTTGCTGCTCATCAACTGTGTATCTTCTCTCCACTTCATTTTCCATATCTGCAAAATGGGAACAACAATACCAGTCTATATCATAAGGCAGTTATGAGTTTAAAGAGAAAT
73908410 73908420 73908430 73908440 73908450 73908460 73908470 73908480 73908490 73908500 73908510
CDX gr...
KLF6
FAC1
TBP-related factors
Ikaros
GGTTTACATTAATGTGCCTTGAAGCACAAGACAGAAATACAATATAAAAAATATGGCTGGGGCCGGGCACGGTGGCTCACGCCTGTAATCCCAGCACTTTGGGAGGCCGAGC
73908520 73908530 73908540 73908550 73908560 73908570 73908580 73908590 73908600 73908610 73908620
ING4
TGGGCGGATCACAAGGTCAGGAGATCGAGACCATCCTGGCTAACACGGTGAAACCCCATCTCTACTAAAAATACAAAAAATTAGCCGGGTGTGGTGGCAGGCGCCTGTAGTC
)08630 73908640 73908650 73908660 73908670 73908680 73908690 73908700 73908710 73908720 73908730
SREBP factors
CCAGCTAGTCGGTAGGCTGAGGCAGGAGAATGGCGTGAACCCGGGAGGCGGAGCTTGCAGTGAGCCGAGATTGTGCCACTGCACTCCAGCCTGGGTGACAGACCGAGACTCC
73908750 73908760 73908770 73908780 73908790 73908800 73908810 73908820 73908830 73908840
ING4
Ikaros
GTCTCAAAAAAAAAAAAAAAAAAAATATGGCTGGGCGTGGTGGCTCATGCCTGTAATCCCAGCACTTTGGAAGGATGAGGTGGGAGGACCCCTTGAACCCAGAAGCCCAGCAA
73908860 73908870 73908880 73908890 73908900 73908910 73908920 73908930 73908940 73908950 73908960
paired...
AACCTTGTCTTTAAAAAAAAAAAAAGAACTGTGCACAAAGATTTCAGAGAGTGCTAAAGATTAGCGCATGGATAAGGAAGTTCTGTGAAGAGTTGAAGTGCTAGGTGAAGAGC
73908970 73908980 73908990 73909000 73909010 73909020 73909030 73909040 73909050 73909060 73909070
FPM315

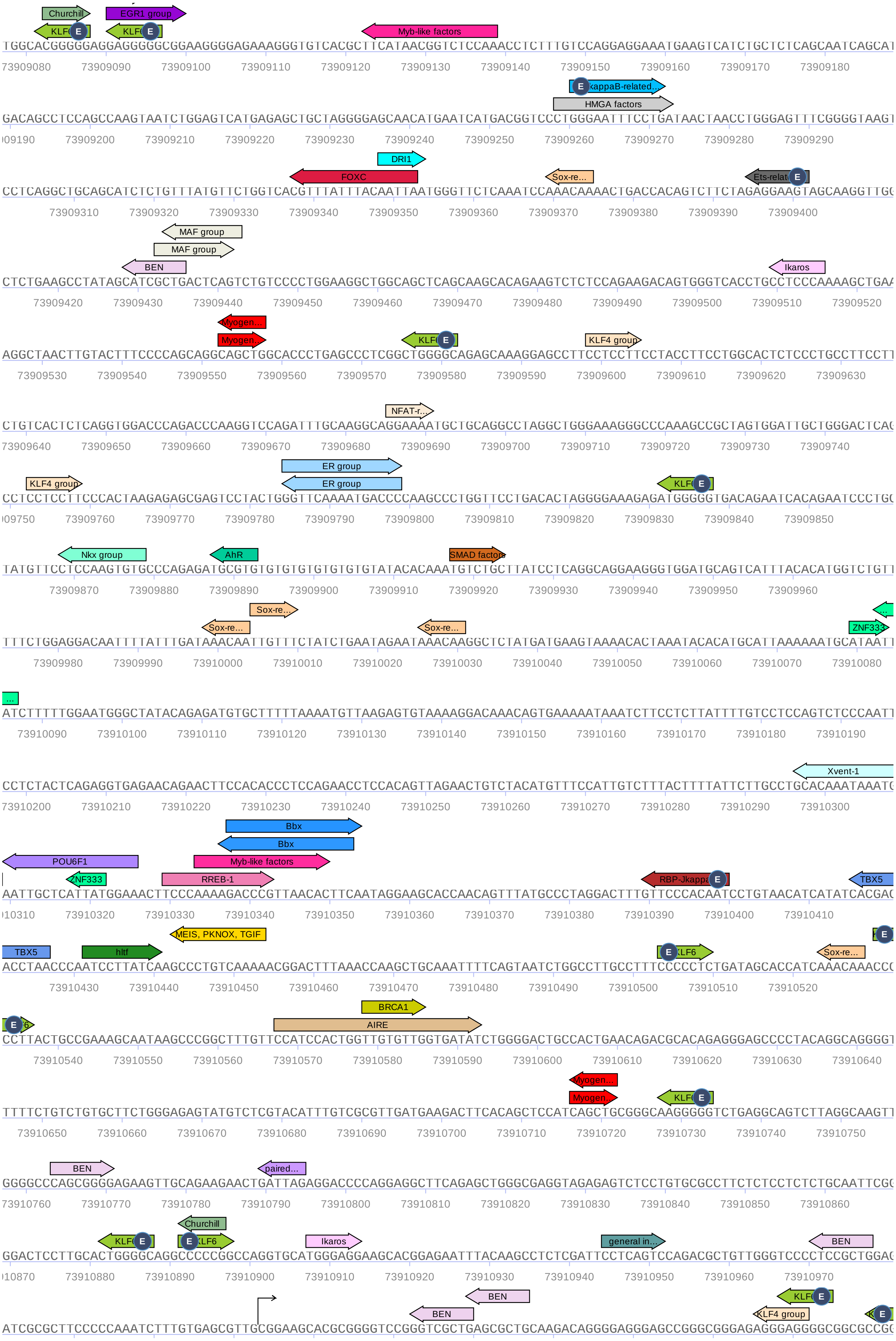

Churchill
EGR1 group
KLF(E
KLF(E
Myb-like factors
TGGCACGGGGGAGGAGGGGGCGGAAGGGGAGAAAGGGTGTCACGCTTCATAACGGTCTCCAAACCTCTTTGTCCAGGAGGAAATGAAGTCATCTGCTCTCAGCAATCAGCAT
73909080 73909090 73909100 73909110 73909120 73909130 73909140 73909150 73909160 73909170 73909180
E kappaB-related...
HMGA factors
GACAGCCTCCAGCCAAGTAATCTGGAGTCATGAGAGCTGCTAGGGGAGCAACATGAATCATGACGGTCCCTGGGAATTTCCTGATAACTAACCTGGGAGTTTCGGGGTAAGT
)09190 73909200 73909210 73909220 73909230 73909240 73909250 73909260 73909270 73909280 73909290
DRI1
FOXC
Sox-re...
Ets-relat E
CCTCAGGCTGCAGCATCTCTGTTTATGTTCTGGTCACGTTTATTTACAATTAATGGGTTCTCAAATCCAAACAAAACTGACCACAGTCTTCTAGAGGAAGTAGCAAGGTTGC
73909310 73909320 73909330 73909340 73909350 73909360 73909370 73909380 73909390 73909400
MAF group
MAF group
BEN
Ikaros
CTCTGAAGCCTATAGCATCGCTGACTCAGTCTGTCCCCTGGAAGGCTGGCAGCTCAGCAAGCACAGAAGTCTCTCCAGAAGACAGTGGGTCACCTGCCTCCCAAAAGCTGAA
73909420 73909430 73909440 73909450 73909460 73909470 73909480 73909490 73909500 73909510 73909520
Myogen...
Myogen
KLF(E
KLF4 group
AGGCTAACTTGTACTTTCCCCAGCAGGCAGCTGGCACCCTGAGCCCTCGGCTGGGGCAGAGCAAAGGAGCCTTCCTCCTTCCTACCTTCCTGGCACTCTCCCTGCCTTCCTT
73909530 73909540 73909550 73909560 73909570 73909580 73909590 73909600 73909610 73909620 73909630
NFAT-r...
CTGTCACTCTCAGGTGGACCCAGACCCAAGGTCCAGATTTGCAAGGCAGGAAAATGCTGCAGGCCTAGGCTGGGAAAGGGCCCAAAGCCGCTAGTGGATTGCTGGGACTCAC
73909640 73909650 73909660 73909670 73909680 73909690 73909700 73909710 73909720 73909730 73909740
ER group
ER group
KLF4 group
KLF(E
CCTCCTCCTTCCCACTAAGAGAGCGAGTCCTACTGGGTTCAAAATGACCCCAAGCCCTGGTTCCTGACACTAGGGGAAAGAGATGGGGGTGACAGAATCACAGAATCCCTGC
)09750 73909760 73909770 73909780 73909790 73909800 73909810 73909820 73909830 73909840 73909850
Nkx group
AhR
SMAD factors
TATGTTCCTCCAAGTGTGCCCAGAGATGCGTGTGTGTGTGTGTGTATACACAAATGTCTGCTTATCCTCAGGCAGGAAGGGTGGATGCAGTCATTTACACATGGTCTGTT
73909870 73909880 73909890 73909900 73909910 73909920 73909930 73909940 73909950 73909960
Sox-re...
Sox-re...
Sox-re...
...
ZNF333
TTTCTGGAGGACAATTTTATTTGATAAACAATTGTTTCTATCTGAATAGAATAAACAAGGCTCTATGATGAAGTAAAACACTAAATACACATGCATTAAAAAATGCATAATT
73909980 73909990 73910000 73910010 73910020 73910030 73910040 73910050 73910060 73910070 73910080
...
ATCTTTTTGGAATGGGCTATACAGAGATGTGCTTTTTAAAATGTTAAGAGTGTAAAAGGACAAACAGTGAAAAATAAATCTTCCTCTTATTTTGTCCTCCAGTCTCCCAATT
73910090 73910100 73910110 73910120 73910130 73910140 73910150 73910160 73910170 73910180 73910190
Xvent-1
CCTCTACTCAGAGGTGAGAACAGAACTTCCACACCCTCCAGAACCTCCACAGTTAGAACTGTCTACATGTTTCCATTGTCTTTACTTTTATTCTTGCCTGCACAAATAAATC
73910200 73910210 73910220 73910230 73910240 73910250 73910260 73910270 73910280 73910290 73910300
Bbx
Bbx
POU6F1
Myb-like factors
ZNF333
RREB-1
RBP-Jkappa E
TBX5
AATTGCTCATTATGGAAACTTCCCAAAAGACCCGTTAACACTTCAATAGGAAGCACCAACAGTTTATGCCCTAGGACTTTGTTCCCACAATCCTGTAACATCATATCACGAC
)10310 73910320 73910330 73910340 73910350 73910360 73910370 73910380 73910390 73910400 73910410
MEIS, PKNOX, TGIF
E
TBX5
hltf
E KLF6
Sox-re...
ACCTAACCCAATCCTTATCAAGCCCTGTCAAAAACGGACTTTAAACCAAGCTGCAAATTTTCAGTAATCTGGCCTTGCCTTTCCCCCTCTGATAGCACCATCAACAAACCC
73910430 73910440 73910450 73910460 73910470 73910480 73910490 73910500 73910510 73910520
BRCA1
E
AIRE
CCTTACTGCCGAAAGCAATAAGCCCGGCTTTGTTCCATCCACTGGTTGTGTTGGTGATATCTGGGGACTGCCACTGAACAGACGCACAGAGGGAGCCCCTACAGGCAGGGGT
73910540 73910550 73910560 73910570 73910580 73910590 73910600 73910610 73910620 73910630 73910640
Myogen...
Myogen
KLF(E
TTTTCTGTCTGTGCTTCTGGGAGAGTATGTCTCGTACATTTGTCGCGTTGATGAAGACTTCACAGCTCCATCAGCTGCGGGCAAGGGGGTCTGAGGCAGTCTTAGGCAAGTT
73910650 73910660 73910670 73910680 73910690 73910700 73910710 73910720 73910730 73910740 73910750
BEN
paired...
GGGGCCCAGCGGGGAGAAGTTGCAGAAGAACTGATTAGAGGACCCCAGGAGGCTTCAGAGCTGGGCGAGGTAGAGAGTCTCCTGTGCGCCTTCTCTCCTCTCTGCAATTCGC
73910760 73910770 73910780 73910790 73910800 73910810 73910820 73910830 73910840 73910850 73910860
Churchill
KLF(E
E KLF6
Ikaros
general in...
BEN
GGACTCCTTGCACTGGGGCAGGCCCCCGGCCAGGTGCATGGGAGGAAGCACGGAGAATTTACAAGCCTCTCGATTCCTCAGTCCAGACGCTGTTGGGTCCCCTCCGCTGGAC
)10870 73910880 73910890 73910900 73910910 73910920 73910930 73910940 73910950 73910960 73910970
BEN
BEN
KLF(E
KLF4 group
E
ATCGCGCTTCCCCCAAATCTTTGTGAGCGTTGCGGAAGCACGCGGGGTCCGGGTCGCTGAGCGCTGCAAGACAGGGGAGGGAGCCGGGCGGGAGAGGGAGGGGCGGCGCCGC

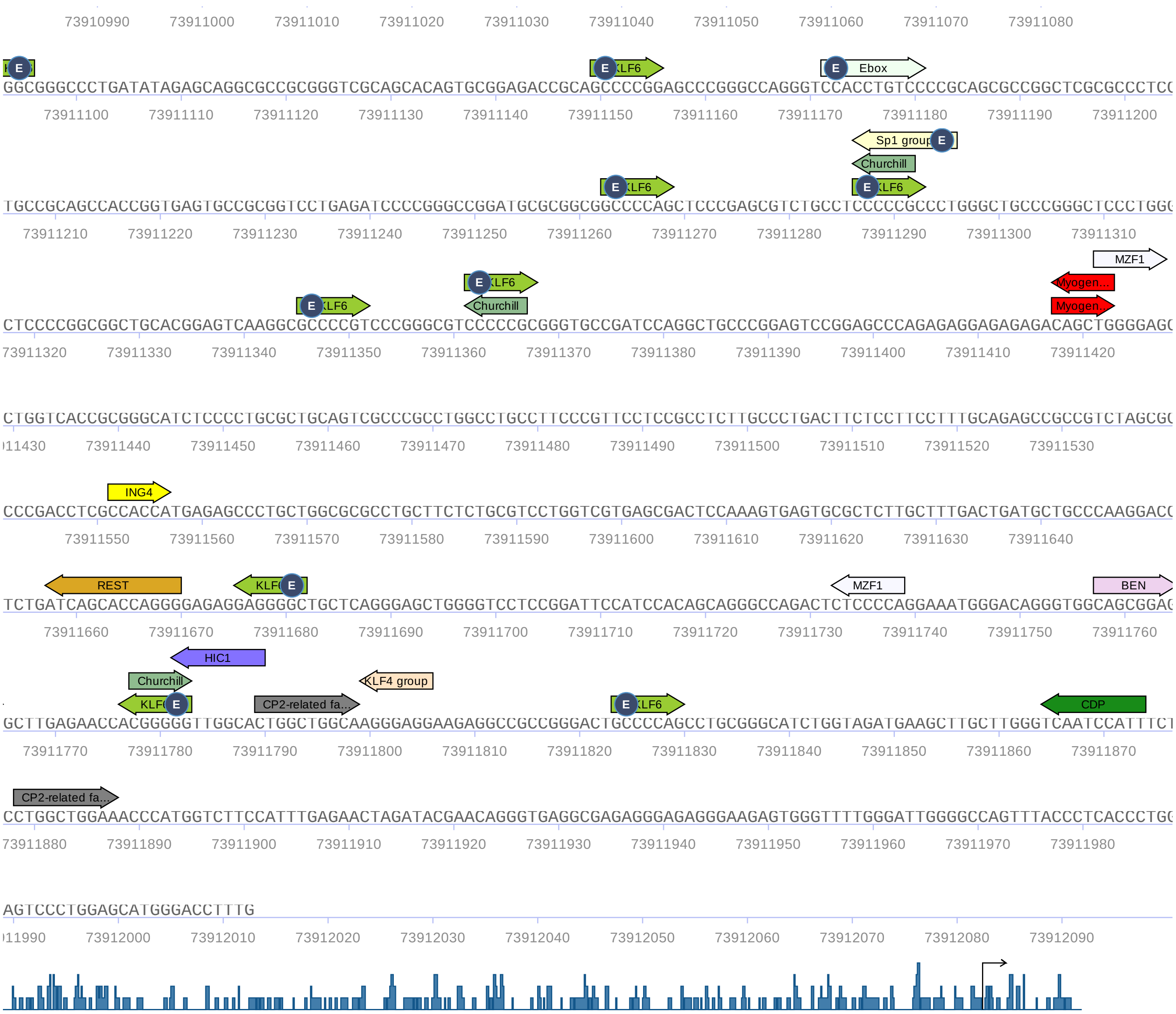
73910990 73911000 73911010 73911020 73911030 73911040 73911050 73911060 73911070 73911080
KLF6 Ebox
GGCGGGCCCTGATATAGAGCAGGCGCCGCGGGTCGCAGCACAGTGCGGAGACCGCAGCCCCGGAGCCCGGGCCAGGGTCCACCTGTCCCCGCAGCGCCGGCTCGCGCCCTCC
73911100 73911110 73911120 73911130 73911140 73911150 73911160 73911170 73911180 73911190 73911200
Sp1 group
Churchill
KLF6 KLF6
TGCCGCAGCCACCGGTGAGTGCCGCGGTCCTGAGATCCCCGGGCCGGATGCGCGGCGGCCCCAGCTCCCGAGCGTCTGCCTCCCCCGCCCTGGGCTGCCCGGGCTCCCTGGG
73911210 73911220 73911230 73911240 73911250 73911260 73911270 73911280 73911290 73911300 73911310
MZF1
KLF6 Myogen...
KLF6 Churchill Myogen...
CTCCCCGGCGGCTGCACGGAGTCAAGGCGCCCCGTCCCGGGCGTCCCCCGCGGGTGCCGATCCAGGCTGCCCGGAGTCCGGAGCCCAGAGAGGAGAGAGACAGCTGGGGAGC
73911320 73911330 73911340 73911350 73911360 73911370 73911380 73911390 73911400 73911410 73911420
CTGGTCACCGCGGGCATCTCCCCTGCGCTGCAGTCGCCCGCCTGGCCTGCCTTCCCGTTCCTCCGCCTCTTGCCCTGACTTCTCCTTCCTTTGCAGAGCCGCCGTCTAGCGC
11430 73911440 73911450 73911460 73911470 73911480 73911490 73911500 73911510 73911520 73911530
ING4
CCCGACCTCGCCACCATGAGAGCCCTGCTGGCGCGCCTGCTTCTCTGCGTCCTGGTCGTGAGCGACTCCAAAGTGAGTGCGCTCTTGCTTTGACTGATGCTGCCCAAGGACC
73911550 73911560 73911570 73911580 73911590 73911600 73911610 73911620 73911630 73911640
REST KLF6 MZF1 BEN
TCTGATCAGCACCAGGGGAGAGGAGGGGCTGCTCAGGGAGCTGGGGTCCTCCGGATTCCATCCACAGCAGGGCCAGACTCTCCCCAGGAAATGGACAGGGTGGCAGCGGAG
73911660 73911670 73911680 73911690 73911700 73911710 73911720 73911730 73911740 73911750 73911760
HIC1
Churchill KLF4 group
KLF6 CP2-related fa... KLF6 CDP
GCTTGAGAACCACGGGGGTTGGCACTGGCTGGCAAGGGAGGAAGAGGCCGCCGGGACTGCCCCAGCCTGCGGGCATCTGGTAGATGAAGCTTGCTTGGGTCAATCCATTTCT
73911770 73911780 73911790 73911800 73911810 73911820 73911830 73911840 73911850 73911860 73911870
CP2-related fa...
CCTGGCTGGAAACCCATGGTCTTCCATTTGAGAACTAGATACGAACAGGGTGAGGCGAGAGGGAGAGGGAAGAGTGGGTTTTGGGATTGGGGCCAGTTTACCCTCACCCTGG
73911880 73911890 73911900 73911910 73911920 73911930 73911940 73911950 73911960 73911970 73911980
AGTCCCTGGAGCATGGGACCTTTG
11990 73912000 73912010 73912020 73912030 73912040 73912050 73912060 73912070 73912080 73912090

Supplementary Figure S4
SERPINE1 Promoter/Enhancer analysis

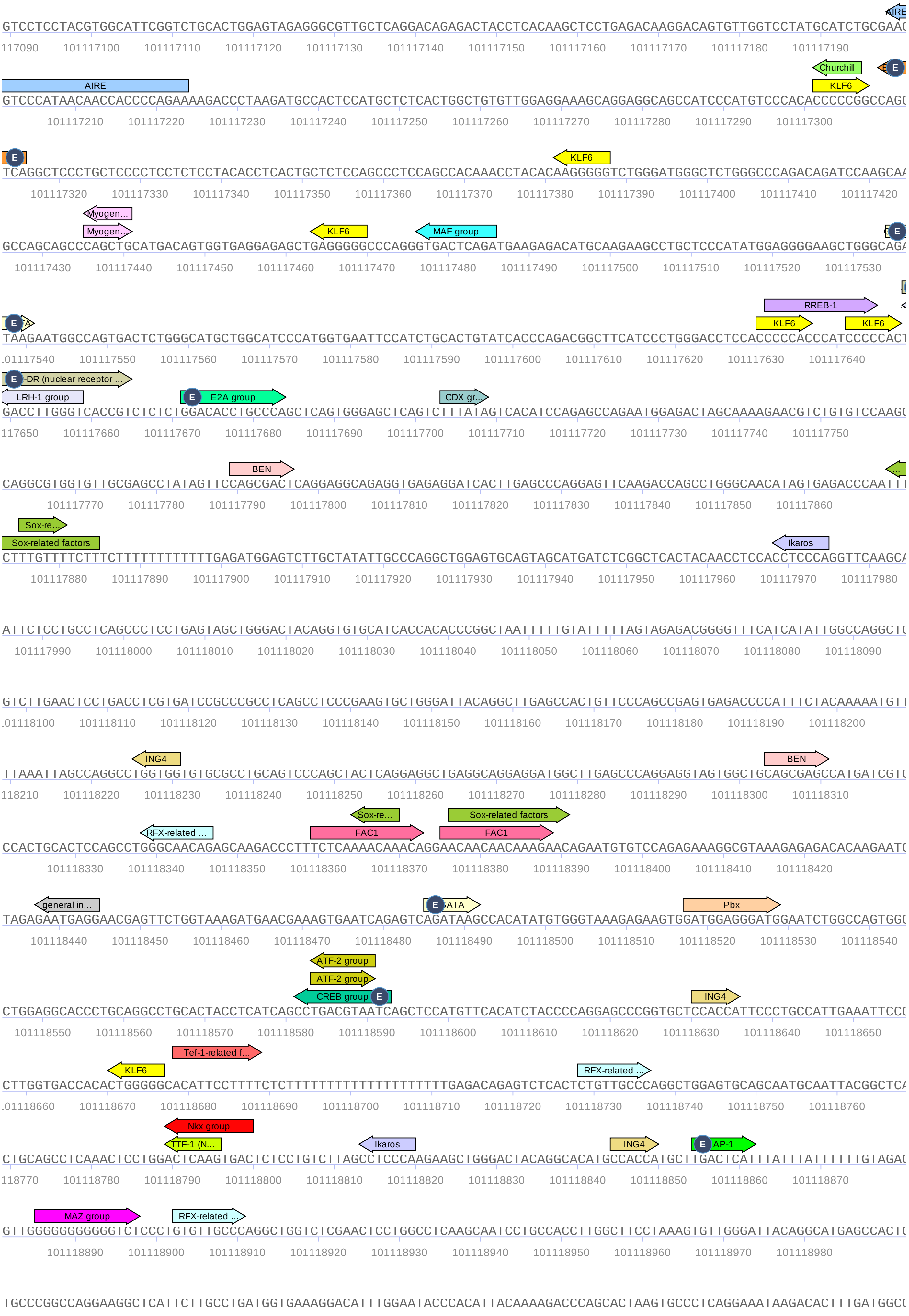

101119000 101119010 101119020 101119030 101119040 101119050 101119060 101119070 101119080 101119090 101119100

CDX gr... | TBP-related factors | Myog... | POU domain group | Myog...

ACATAAGTTTAAATACAGAAATAACTGTAAGACAAGGGAGAATGCAAATATATAAAAGTGATAAAAGCTAAAAATTAGCTGGGTGTGGTCGTGCATGCCTGTGGTCCCAGCT

101119110 101119120 101119130 101119140 101119150 101119160 101119170 101119180 101119190 101119200 101119210

... | Sox-re... | SREBP factors

GCTCAGGAGGCTGAGGTGAGAGGATTGTTTGAGCCCAGGAGTTGGAGGCTGCAGTGAGCCATGATCACACAACTGCACTCCAGCCTGGGCAACAAAGTGAGACCCTATCTTA

.01119220 101119230 101119240 101119250 101119260 101119270 101119280 101119290 101119300 101119310 101119320

AAAAAAAAAAAAAAAAATAGACTGGGCGCGGTGGCTCATGCCTGTAAACCCAGCACTTCGGGAAGCTGAGGTGGGAAGATCAGTTGAGCTCAGGAGTTTGAGACCAGCCTGG

119330 101119340 101119350 101119360 101119370 101119380 101119390 101119400 101119410 101119420 101119430

p53 related factors E | E p53 related factors | p53 related factors

CCAACATGGTGAAACCCTGTCTCTACTAAAAATCCAAAAATTAGTTGGGCATGTTTGTGCAGGCCTGTAGTCCCTGCTACTCAGGAGGCTGAGGCACGAGAATTGCTTGAAC

101119450 101119460 101119470 101119480 101119490 101119500 101119510 101119520 101119530 101119540

E ER group

CCGTGAGGTGGAGGTTGCAGTGAGCTGAGATCGCGTCACTGCATTCTAGCTTGGGTGACTGAGTGAGACTCTGTCTCAAAAAAAAAAAAAAGATAAAATTGGCTATGGTAGAG

101119560 101119570 101119580 101119590 101119600 101119610 101119620 101119630 101119640 101119650 101119660

CDP | paired... | ZNF333 | CDP | KLF6

CATACATTTTTTTTAAGTGATTAAAGCATTATAGGTGCCATAGAGGAATCGATGAGAGATGGGAGATGGAGGCTGCAGGGGCCTAGCCTGGAAGAGTGGGTCGAGCTCAACT

101119670 101119680 101119690 101119700 101119710 101119720 101119730 101119740 101119750 101119760 101119770

YY1-like... | KLF4 group | E group...

CCATTTTGGTAAATCAAGTCTCCTTCCTCCCTGGTATGCTCAGAAAATAAGGATACACTTGGGACAAAAGCGGGGTACATCCATGACCTGCAGCCCAGAGGGGAATATCTTC

.01119780 101119790 101119800 101119810 101119820 101119830 101119840 101119850 101119860 101119870 101119880

MAF group | KLF6

CTGGGATCTGAAACCTGCAAAGTCAGCCAATGGGAGCGTGTCAGGACCAGGGAGACAGCAGAAGGATCTGGTATGAAACAGATGAGAGGGGCTGCCAGGCATCCCCTCGGGT

119890 101119900 101119910 101119920 101119930 101119940 101119950 101119960 101119970 101119980 101119990

Sox-re... | Pbx | ZNF333

AAGCAGCCAGCCCACTTCCGAGGCATTTAACGCCCACGCTCCGCTAAATGCTGAGAGACGTAAAAACAAACACCTCAAACATCCATCCCTCAAGGAGCTTGCATTATATCAA

101120010 101120020 101120030 101120040 101120050 101120060 101120070 101120080 101120090 101120100

ING4

AATGAACAGCTCAGACTGTCTGGGTTCCTGTCCTGGCTCTGCTGCCCACCAAGAGCGGCTGCCACAAAGGAGAACATTTGTTCTTGCCAAATGTGTTGGACATTTGGCAGAT

101120120 101120130 101120140 101120150 101120160 101120170 101120180 101120190 101120200 101120210 101120220

ATF-2 group | ATF-2 group | KL...

GACGTTTTATGTAATTCTCATTGCAACCTTGAAAGATGGGAATCGTTATCTGTCTTTTACAAATAAGAATACTGAGGCTGTGATCCCTAAATCATAGGGAAGGAAGGAAGGG

101120230 101120240 101120250 101120260 101120270 101120280 101120290 101120300 101120310 101120320 101120330

KLF6 | KLF4 group | K... | MAZ | paired...

AGGGAGGGGGAGGGGAGGAAGAGGAGGAGGAAGAGAAGGAGAAGGAGAAGGGATTAAGTTTTACCTAGTCACATAGCCAATGTCAGATTCCTAACTAGTGGCCGGGTCTGTC

.01120340 101120350 101120360 101120370 101120380 101120390 101120400 101120410 101120420 101120430 101120440

Nkx group | general in... | E RI1

TGATCCAATGATCACTATTCTCTCATTTATGGTGTAGTCACTGTGTGGCTTCAACCACAGTGGACCTCTCTGGACCTAAGTGCCCTCACTTGTAAATTAAAAGAACTGGGTT

120450 101120460 101120470 101120480 101120490 101120500 101120510 101120520 101120530 101120540 101120550

ING4 | Nkx group | p53 related factors E | Ikaros | NR-DR (nuclear receptor d E

AGGGCCAGGCATGGTGGCTCATGCCTGTAACCACAGCACTTTGGGAGGCTGAGGCAGGTCGGTCACTTGAGCTCAGGAGTTCAAGAACAGCCTGGGCAACGTGGCAAAACCC

101120570 101120580 101120590 101120600 101120610 101120620 101120630 101120640 101120650 101120660

MECP2 | TBX5 | Ikaros | Ikaros | paired...

CGTCTCTACCAAAAATACAAAAAATTAGCCAGGTGTCATGGTGTACATCTGTGGTCCCAGCTACTGGGAGGCTGAGGTGGGAGGATCACTTAATCCCGGGAGGTAGAGGCTG

101120680 101120690 101120700 101120710 101120720 101120730 101120740 101120750 101120760 101120770 101120780

BRCA1 | KLF6

CAGTAAGCTGAAATGGCACCACTGCACTCCAACCTTGAGCCCAGGAGTTTGAGACTAGCCTGGGCAACATAAAGAGACCCTCATCTCTGAAAAAAAAAAAAATTAGGCGGGG

101120790 101120800 101120810 101120820 101120830 101120840 101120850 101120860 101120870 101120880 101120890

Nkx group | NR-DR (nuclear receptor d E

ACAGTGGCTCACATCTGTAATCCCAGCACTTTGAGAGGCTGAGGAAGGCGGATCACTTGAGGTCAGGAGTTCAAGACCAGCCTGGCCAACATGGTGAAACCCCGTCTCTACT

.01120900 101120910 101120920 101120930 101120940 101120950 101120960 101120970 101120980 101120990 101121000

Myogen... | Myogen... | Ikaros | ZNF333 | Ikaros | E ER group

AAAAATACAAAAATCAGCTGGGCGTGATGGCAGATGCCTGTAATCCCAGCTACTTGGGAGGCTGAAGCACTATAATTGCTTGAGCCTGGGAGGTAGAGGTTGCAGTGAACTG

121010 101121020 101121030 101121040 101121050 101121060 101121070 101121080 101121090 101121100 101121110

ING4 KLF6

AGACCACACCATTGCACTCCAGCCTGGGCAAGAAGAGCGAAACTTTGTCTCAAAAAAAAAAAAAAAAAAAAAAAAAAATTGGCGGGGCATGGTGGTGCACACCTGTAGTCCC

101121130 101121140 101121150 101121160 101121170 101121180 101121190 101121200 101121210 101121220

NF-1A Ikaros Ikaros MAF group

AACTAGCAACTCCTCAGGAGGCTGAGATGGGAGGATCTCTTGAGCCTGGGAGGTCGAAATGCAGTGAGTCATGACTGTGCCAAGGCTGGGAGCCATGATTGTGCCACTGCAC

101121240 101121250 101121260 101121270 101121280 101121290 101121300 101121310 101121320 101121330 101121340

BRCA1 RFX-related ... KLF6 HMGA factors

TCCCACCTGGGCAACAGAACGTGGCCCCCACTCAAAAAAAAAATGCAATTGTGGACAAGCAAGGGCTACATTGGGTAAGGCAGCACCAGTTAGAGTGTTAGGGAGTTTAAAG

101121350 101121360 101121370 101121380 101121390 101121400 101121410 101121420 101121430 101121440 101121450

KLF6 KLF6 KLF6

AGAAATGACCGGCCAGGGGCTGGCAGGGGAGAGGGGATACAGCAGGAGAGGAGGGGGAAGTCAGTCACAGAGCCAGGGTAAGGAAGGGCCCCTGGACAGGCTGTCCCAGAGC

.01121460 101121470 101121480 101121490 101121500 101121510 101121520 101121530 101121540 101121550 101121560

ZNF333

CACGATCTTAAATAAAAGGCAGTGGAGGGCCAGGCGCAGTGGCTCACGCCTATAATCCCAGCACTTTGGAAGGCCGAGGTGGGTGGATCATGAGGTCAGGAGATCGAGACCA

121570 101121580 101121590 101121600 101121610 101121620 101121630 101121640 101121650 101121660 101121670

ING4 ZNF333 p53 related factors E KLF6

GCCTGGACAACATGGGGAAACTAGTCTCTACTAAAAATACAAAAATTAGCCAGGCATGGTGGCGCATGACTATAATCCCAGCTACTCGGGAGGCTGGGGCAGGAGAATCGCT

101121690 101121700 101121710 101121720 101121730 101121740 101121750 101121760 101121770 101121780

Ikaros TBX5 RFX-related ...

TGAACCTGGGAGGCGGAGGATGCAGTGAGCCAAGATCACACCTCCGCACTCCAGCCTGGGCAACAGAGCAAGACTCCATCTCAAAAAAAAAAAAAAAAAAGGCGGTGGAGAC

101121800 101121810 101121820 101121830 101121840 101121850 101121860 101121870 101121880 101121890 101121900

Sox-re... BRCA1

ACCCAGCACTGGTTCCCTCCTAGAGAAGGGGAGAGAGCCGAGCCCAAACACAGGCTATTTTTGGAGTTGTGTTGTTTGGAGAGCAACCAAGCCAGAAATAGTGCTGTTAGCA

101121910 101121920 101121930 101121940 101121950 101121960 101121970 101121980 101121990 101122000 101122010

Churchill Tef-1-related f... KLF6 KLF4 group KLF6

AGACCCTGCCTTCTCCAACCCCCGGCTCTCCCAGTGAAATCACCAGGAATCAGGGAGGCTGGGCCCCACATTCCAGGGAGCCTGTGTAACAGGCAGTGCTCTCTTCCCCACC

.01122020 101122030 101122040 101122050 101122060 101122070 101122080 101122090 101122100 101122110 101122120

MAF group KLF6

GGAAGGGCTTGGGAGCTGAGCATTTGACTGTAAAAGGGATTCTGGGATTCCAGAGGGACAGAGGGACTGAGTCAGAGAGAGAGAGAGAGAGAGTGGAAATGGGGGGCAAC

122130 101122140 101122150 101122160 101122170 101122180 101122190 101122200 101122210 101122220 101122230

Blimp-1 BEN IRF factors

CTCCTGCAGCGGAGGAGAGGGGACAGGAGGGAAGGCTCGGGGAAGAAAGGGTAGGCAGGAGGTCAGAGAGAAGGGAGCAGGAAGCGGGAGGCTCAGAGAGAGAAAGGAAAG

101122250 101122260 101122270 101122280 101122290 101122300 101122310 101122320 101122330 101122340

ING4 KLF6 GLI group GLI group ...

TGAAAAAGAAAGCGGGGAAGACAGAAGCAAAGAGGAAGAGCAGAGATCGGGCCAGAAGGCGGGAGGTCAGGGGGCGATGGGTGGTGGGAAGGCCAGTGTCCACCCAGGGCCC

101122360 101122370 101122380 101122390 101122400 101122410 101122420 101122430 101122440 101122450 101122460

GLI group ING4 KLF6 NFAT-r...

ACCAGTGGGTGGGGGACAGAGGGACAGTCATGGAAAATCTGAGTGTGATCTACAGTCACGAACCACCTGCTCAGGGACAGATGGAGACATCTTTCCCAGTAGCTGGAATCAG

101122470 101122480 101122490 101122500 101122510 101122520 101122530 101122540 101122550 101122560 101122570

GAT E

AACTTTCCCCTGAAATCCACAGCCCGGTAACTCACTCTCTTATCTCTGACCCTCCGGAAGCCAGAGATCTAGCTATTTCATCAGCATGGACTTTTCTTTCTGTGCCCGTGTG

.01122580 101122590 101122600 101122610 101122620 101122630 101122640 101122650 101122660 101122670 101122680

NFAT-r... TTF-1 (N...

ACCCCTTACAAAAGAGCTCAAGCTGGAAAGAGAGGATTGGATCAAGAAAGAGAGAACTTAGGATTCAGGGGAAAAGGGGAAAGTGATAACCCCTTGAGTCAGATTCTGAGAA

122690 101122700 101122710 101122720 101122730 101122740 101122750 101122760 101122770 101122780 101122790

Myogen... E MZF1 Myogen...

GAGGGTTTCTCTGAGAGGTCGGTGGGGAGCAGCCCGCAGGCAGCTGGACCCTGGTATGGAAGGGCTCGGCTCCTAAGGCAGGTAAGAGAAGGCAGGGCCACGTGCATGAAGT

101122810 101122820 101122830 101122840 101122850 101122860 101122870 101122880 101122890 101122900

NF-1 factors E

AGAGGCAGGGGTCATGGCCAAGTCTGGGTTCTTTTTATTTATTTATTTTTTTGAGACAATCTTGCTCTTTCGCTCAAGCTGGAGTGCAGTGGTGCAATCTCAGCTCTCTGCA

101122920 101122930 101122940 101122950 101122960 101122970 101122980 101122990 101123000 101123010 101123020

E HIF1

ACCTCCACCTCTTGGGTTCAAGAGATTCTCCCACCTCAGGCTCCTGAGTAGCTGGGACTACAGGTACGTGCCGCCATGCCTGGCTAATTTCTGTACTTTTTTTTTTTTTAGTA

101123030 101123040 101123050 101123060 101123070 101123080 101123090 101123100 101123110 101123120 101123130

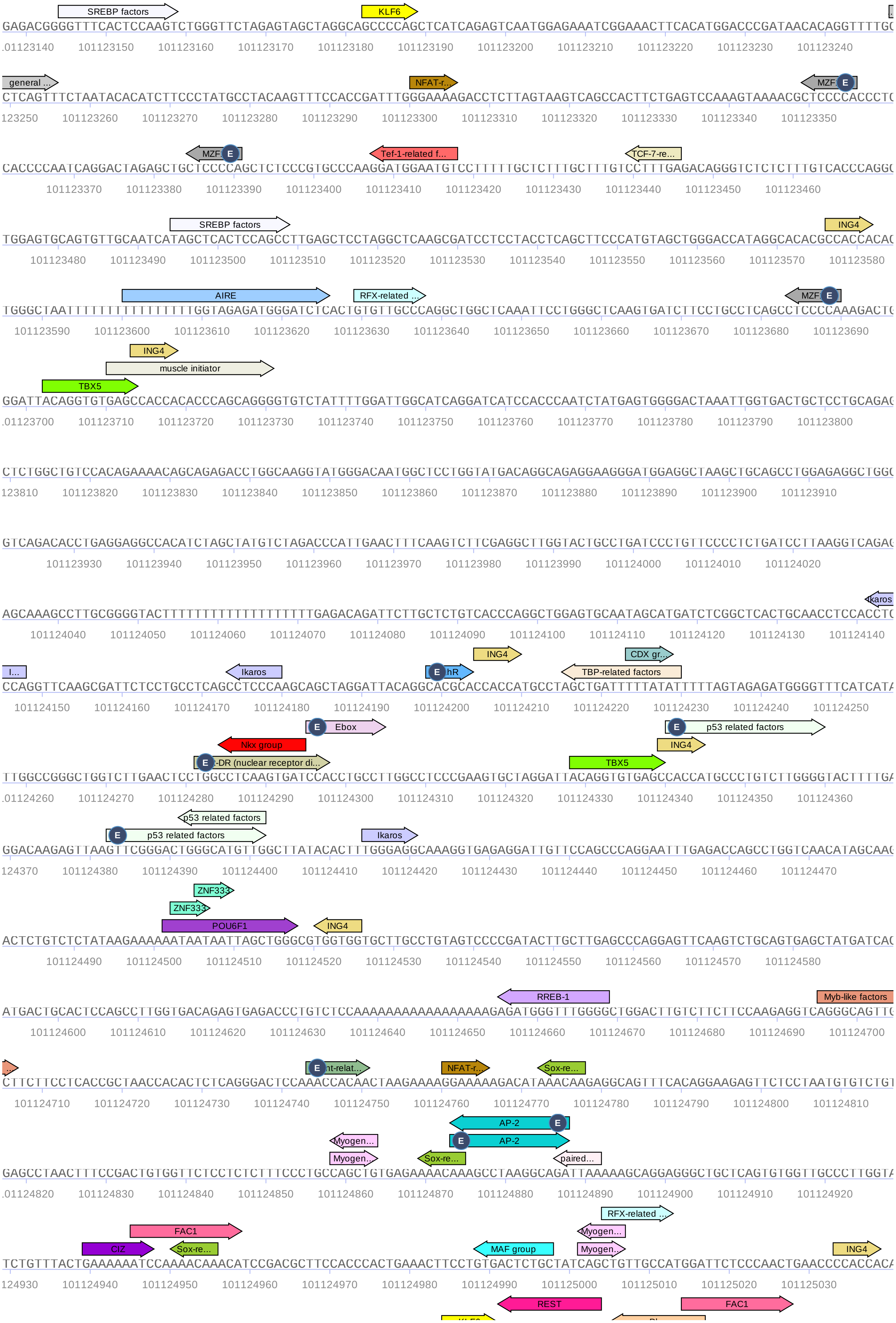
SREBP factors
KLF6
GAGACGGGGTTTCACTCCAAGTCTGGGTTCTAGAGTAGCTAGGCAGCCCCAGCTCATCAGAGTCAATGGAGAAATCGGAAACTTCACATGGACCCGATAACACAGGTTTTGC
.01123140 101123150 101123160 101123170 101123180 101123190 101123200 101123210 101123220 101123230 101123240
general ...
NFAT-r...
MZF E
CTCAGTTTCTAATACACATCTTCCCTATGCCTACAAGTTTCCACCGATTTGGGAAAAGACCTCTTAGTAAGTCAGCCACTTCTGAGTCCAAAGTAAAACGCTCCCCACCCTC
123250 101123260 101123270 101123280 101123290 101123300 101123310 101123320 101123330 101123340 101123350
MZF E
Tef-1-related f...
TCF-7-re...
CACCCCAATCAGGACTAGAGCTGCTCCCCAGCTCTCCCGTGCCCAAGGATGGAATGTCCTTTTTGCTCTTTGCTTTGTCCTTTGAGACAGGGTCTCTCTTTGTCACCCAGGC
101123370 101123380 101123390 101123400 101123410 101123420 101123430 101123440 101123450 101123460
SREBP factors
ING4
TGGAGTGCAGTGTTGCAATCATAGCTCACTCCAGCCTTGAGCTCCTAGGCTCAAGCGATCCTCCTACCTCAGCTTCCCATGTAGCTGGGACCATAGGCACACGCCACCACAC
101123480 101123490 101123500 101123510 101123520 101123530 101123540 101123550 101123560 101123570 101123580
AIRE
RFX-related ...
MZF E
TGGGCTAATTTTTTTTTTTTTTTTTTGGTAGAGATGGGATCTCACTGTGTTGCCCAGGCTGGCTCAAATTCCTGGGCTCAAGTGATCTTCCTGCCTCAGCCTCCCCAAAGACTG
101123590 101123600 101123610 101123620 101123630 101123640 101123650 101123660 101123670 101123680 101123690
ING4
muscle initiator
TBX5
GGATTACAGGTGTGAGCCACCACACCCAGCAGGGGTGTCTATTTTGGATTGGCATCAGGATCATCCACCCAATCTATGAGTGGGGACTAAATTGGTGACTGCTCCTGCAGAG
.01123700 101123710 101123720 101123730 101123740 101123750 101123760 101123770 101123780 101123790 101123800
CTCTGGCTGTCCACAGAAAACAGCAGAGACCTGGCAAGGTATGGGACAATGGCTCCTGGTATGACAGGCAGAGGAAGGGATGGAGGCTAAGCTGCAGCCTGGAGAGGCTGGC
123810 101123820 101123830 101123840 101123850 101123860 101123870 101123880 101123890 101123900 101123910
GTCAGACACCTGAGGAGGCCACATCTAGCTATGTCTAGACCCATTGAACTTTCAAGTCTTCGAGGCTTGGTACTGCCTGATCCCTGTTCCCCTCTGATCCTTAAGGTCAGAC
101123930 101123940 101123950 101123960 101123970 101123980 101123990 101124000 101124010 101124020
Ikaros
AGCAAAGCCTTGCGGGGTACTTTTTTTTTTTTTTTTTTTGAGACAGATTCTTGCTCTGTCACCCAGGCTGGAGTGCAATAGCATGATCTCGGCTCACTGCAACCTCCACCTC
101124040 101124050 101124060 101124070 101124080 101124090 101124100 101124110 101124120 101124130 101124140
ING4
CDX gr...
I...
Ikaros
E hR
TBP-related factors
CCAGGTTCAAGCGATTCTCCTGCCTCAGCCTCCCAAGCAGCTAGGATTACAGGCACGCACCACCATGCCTAGCTGATTTTTATATTTTTAGTAGAGATGGGGTTTCATCATA
101124150 101124160 101124170 101124180 101124190 101124200 101124210 101124220 101124230 101124240 101124250
E Ebox
E p53 related factors
Nkx group
ING4
E -DR (nuclear receptor di...
TBX5
TTGGCCGGGCTGGTCTTGAACTCCTGGCCTCAAGTGATCCACCTGCCTTGGCCTCCCGAAGTGCTAGGATTACAGGTGTGAGCCACCATGCCCTGTCTTGGGGTACTTTTGA
.01124260 101124270 101124280 101124290 101124300 101124310 101124320 101124330 101124340 101124350 101124360
p53 related factors
E p53 related factors
Ikaros
GGACAAGAGTTAAGTTCGGGACTGGGCATGTTGGCTTATACACTTTGGGAGGCAAAGGTGAGAGGATTGTTCCAGCCCAGGAATTTGAGACCAGCCTGGTCAACATAGCAAG
124370 101124380 101124390 101124400 101124410 101124420 101124430 101124440 101124450 101124460 101124470
ZNF333
ZNF333
POU6F1
ING4
ACTCTGTCTCTATAAGAAAAAATAATAATTAGCTGGGCGTGGTGGTGCTTGCCTGTAGTCCCCGATACTTGCTTGAGCCCAGGAGTTCAAGTCTGCAGTGAGCTATGATCAC
101124490 101124500 101124510 101124520 101124530 101124540 101124550 101124560 101124570 101124580
RREB-1
Myb-like factors
ATGACTGCACTCCAGCCTTGGTGACAGAGTGAGACCCTGTCTCCAAAAAAAAAAAAAAAAAAGAGATGGGTTTGGGGCTGGACTTGTCTTCTTCCAAGAGGTCAGGGCAGTTC
101124600 101124610 101124620 101124630 101124640 101124650 101124660 101124670 101124680 101124690 101124700
...
E nt-relat...
NFAT-r...
Sox-re...
CTTCTTCCTCACCGCTAACCACACTCTCAGGGACTCCAAACCACAACTAAGAAAAGGAAAAAGACATAAACAAGAGGCAGTTTCACAGGAAGAGTTCTCCTAATGTGTCTGT
101124710 101124720 101124730 101124740 101124750 101124760 101124770 101124780 101124790 101124800 101124810
AP-2 E
Myogen...
E AP-2
Myogen...
Sox-re...
paired...
GAGCCTAACTTTCCGACTGTGGTTCTCCTCTCTTTCCCTGCCAGCTGTGAGAAAACAAAGCCTAAGGCAGATTAAAAAGCAGGAGGGCTGCTCAGTGTGGTTGCCCTTGGTA
.01124820 101124830 101124840 101124850 101124860 101124870 101124880 101124890 101124900 101124910 101124920
RFX-related ...
FAC1
Myogen...
CIZ
Sox-re...
MAF group
Myogen...
ING4
TCTGTTTACTGAAAAAATCCAAAACAAACATCCGACGCTTCCACCCACTGAAACTTCCTGTGACTCTGCTATCAGCTGTTGCCATGGATTCTCCCAACTGAACCCCACACA
124930 101124940 101124950 101124960 101124970 101124980 101124990 101125000 101125010 101125020 101125030
REST
FAC1

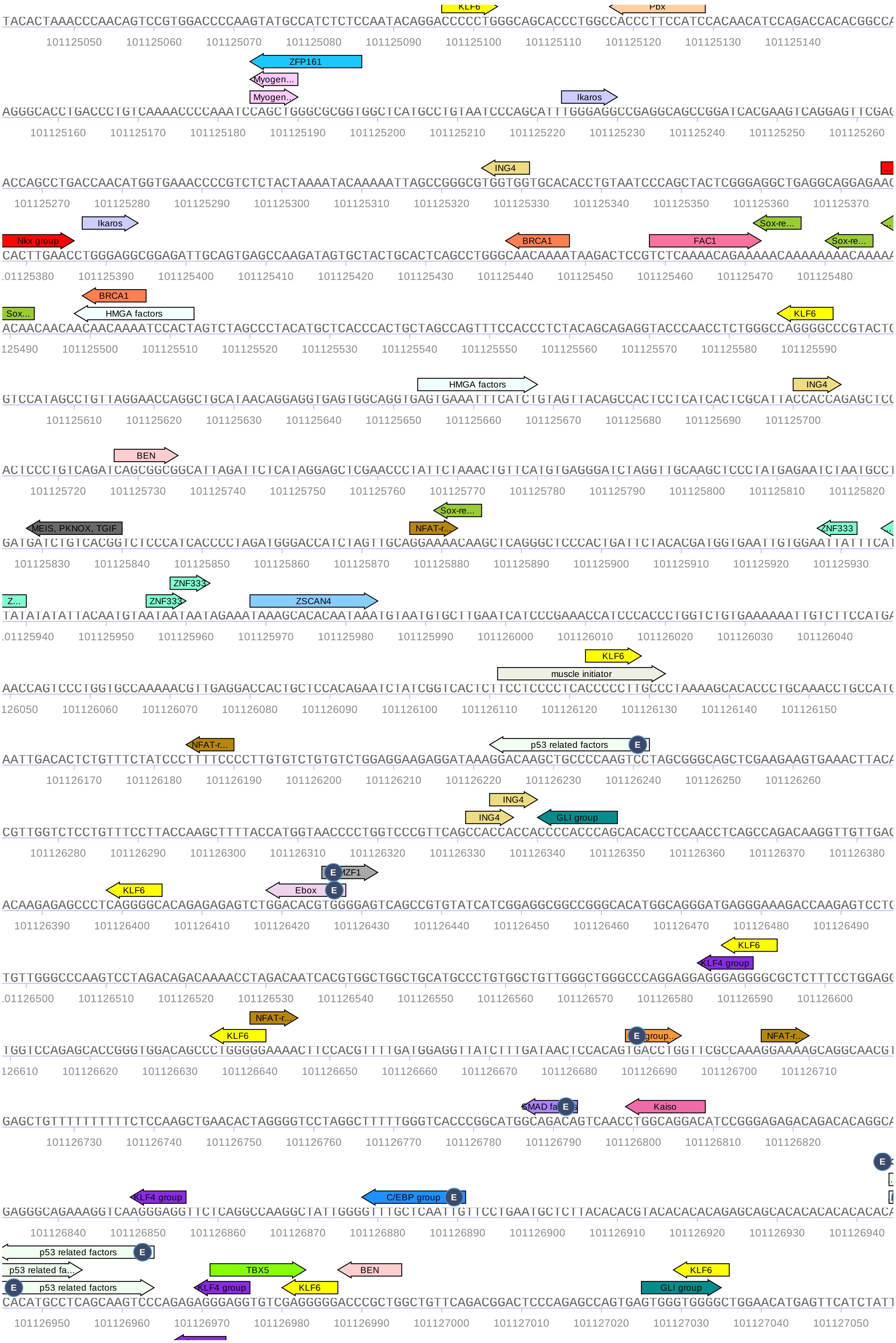
KLF6
Pbx
TACACTAAACCCAACAGTCCGTGGACCCCAAGTATGCCATCTCTCCAATACAGGACCCCCTGGGCAGCACCCTGGCCACCCTTCCATCCACAACATCCAGACCACACGGCCA
101125050 101125060 101125070 101125080 101125090 101125100 101125110 101125120 101125130 101125140
ZFP161
Myogen...
Myogen...
Ikaros
AGGGCACCTGACCCTGTCAAAACCCCAAATCCAGCTGGGCGCGGTGGCTCATGCCTGTAATCCCAGCATTTGGGAGGCCGAGGCAGCCGGATCACGAAGTCAGGAGTTCGAG
101125160 101125170 101125180 101125190 101125200 101125210 101125220 101125230 101125240 101125250 101125260
ING4
...
ACCAGCCTGACCAACATGGTGAAACCCCGTCTCTACTAAAATACAAAAATTAGCCGGGCGTGGTGGTGCACACCTGTAATCCCAGCTACTCGGGAGGCTGAGGCAGGAGAAC
101125270 101125280 101125290 101125300 101125310 101125320 101125330 101125340 101125350 101125360 101125370
Ikaros
Sox-re...
Nkx group
BRCA1
FAC1
Sox-re...
CACTTGAACCTGGGAGGCGGAGATTGCAGTGAGCCAAGATAGTGCTACTGCACTCAGCCTGGGCAACAAAATAAGACTCCGTCTCAAAACAGAAAAACAAAAAAAACAAAAA
.01125380 101125390 101125400 101125410 101125420 101125430 101125440 101125450 101125460 101125470 101125480
BRCA1
Sox...
HMGA factors
KLF6
ACAACAACAACAACAAAATCCACTAGTCTAGCCCTACATGCTCACCCACTGCTAGCCAGTTTCCACCCTCTACAGCAGAGGTACCCAACCTCTGGGCCAGGGGCCCGTACTG
125490 101125500 101125510 101125520 101125530 101125540 101125550 101125560 101125570 101125580 101125590
HMGA factors
ING4
GTCCATAGCCTGTTAGGAACCAGGCTGCATAACAGGAGGTGAGTGGCAGGTGAGTGAAATTTCATCTGTAGTTACAGCCACTCCTCATCACTCGCATTACCACCAGAGCTCC
101125610 101125620 101125630 101125640 101125650 101125660 101125670 101125680 101125690 101125700
BEN
ACTCCCTGTCAGATCAGCGGCGGCATTAGATTCTCATAGGAGCTCGAACCCTATTCTAAACTGTTCATGTGAGGGATCTAGGTTGCAAGCTCCCTATGAGAATCTAATGCCT
101125720 101125730 101125740 101125750 101125760 101125770 101125780 101125790 101125800 101125810 101125820
Sox-re...
MEIS, PKNOX, TGIF
NFAT-r...
ZNF333
GATGATCTGTCACGGTCTCCCATCACCCCTAGATGGGACCATCTAGTTGCAGGAAAACAAGCTCAGGGCTCCCACTGATTCTACACGATGGTGAATTGTGGAATTATTTCAT
101125830 101125840 101125850 101125860 101125870 101125880 101125890 101125900 101125910 101125920 101125930
ZNF333
Z...
ZNF333
ZSCAN4
TATATATATTACAATGTAATAATAATAGAAATAAAGCACACAATAAATGTAATGTGCTTGAATCATCCCGAAACCATCCCACCCTGGTCTGTGAAAAAATTGTCTTCCATGA
.01125940 101125950 101125960 101125970 101125980 101125990 101126000 101126010 101126020 101126030 101126040
KLF6
muscle initiator
AACCAGTCCCTGGTGCCAAAAACGTTGAGGACCACTGCTCCACAGAATCTATCGGTCACTCTTCCTCCCCTCACCCCCTTGCCCTAAAGCACACCCTGCAAACCTGCCATG
126050 101126060 101126070 101126080 101126090 101126100 101126110 101126120 101126130 101126140 101126150
NFAT-r...
p53 related factors
E
AATTGACACTCTGTTTCTATCCCTTTTCCCCTTGTGTCTGTGTCTGGAGGAAGAGGATAAAGGACAAGCTGCCCCAAGTCCTAGCGGGCAGCTCGAAGAAGTGAAACTTACA
101126170 101126180 101126190 101126200 101126210 101126220 101126230 101126240 101126250 101126260
ING4
ING4
GLI group
CGTTGGTCTCCTGTTTCCTTACCAAGCTTTTACCATGGTAACCCCTGGTCCCGTTCAGCCACCACCACCCCACCCAGCACACCTCCAACCTCAGCCAGACAAGGTTGTTGAC
101126280 101126290 101126300 101126310 101126320 101126330 101126340 101126350 101126360 101126370 101126380
E MZF1
KLF6
Ebox E
ACAAGAGAGCCCTCAGGGGCACAGAGAGAGTCTGGACACGTGGGGAGTCAGCCGTGTATCATCGGAGGCGGCCGGGCACATGGCAGGGATGAGGGAAAGACCAAGAGTCCTG
101126390 101126400 101126410 101126420 101126430 101126440 101126450 101126460 101126470 101126480 101126490
KLF6
KLF4 group
TGTTGGGCCCAAGTCCTAGACAGACAAAACCTAGACAATCACGTGGCTGGCTGCATGCCCTGTGGCTGTTGGGCTGGGCCCAGGAGGAGGGAGGGGCGCTCTTTCCTGGAGG
.01126500 101126510 101126520 101126530 101126540 101126550 101126560 101126570 101126580 101126590 101126600
NFAT-r...
KLF6
E group...
NFAT-r...
TGGTCCAGAGCACCGGGTGGACAGCCCTGGGGGAAAACTTCCACGTTTTGATGGAGGTTATCTTTGATAACTCCACAGTGACCTGGTTCGCCAAAGGAAAAGCAGGCAACGT
126610 101126620 101126630 101126640 101126650 101126660 101126670 101126680 101126690 101126700 101126710
SMAD fa E
Kaiso
GAGCTGTTTTTTTTTTCTCCAAGCTGAACACTAGGGGTCCTAGGCTTTTTGGGTCACCCGGCATGGCAGACAGTCAACCTGGCAGGACATCCGGGAGAGACAGACACAGGCA
101126730 101126740 101126750 101126760 101126770 101126780 101126790 101126800 101126810 101126820
E
KLF4 group
C/EBP group E
GAGGGCAGAAAGGTCAAGGGAGGTTCTCAGGCCAAGGCTATTGGGGTTTGCTCAATTGTTCCTGAATGCTCTTACACACGTACACACACAGAGCAGCACACACACACACACA
101126840 101126850 101126860 101126870 101126880 101126890 101126900 101126910 101126920 101126930 101126940
p53 related factors E
p53 related fa...
TBX5
BEN
KLF6
E p53 related factors
KLF4 group
KLF6
GLI group
CACATGCCTCAGCAAGTCCCAGAGAGGGAGGTGTCGAGGGGGACCCGCTGGCTGTTCAGACGGACTCCCAGAGCCAGTGAGTGGGTGGGGCTGGAACATGAGTTCATCTATT
101126950 101126960 101126970 101126980 101126990 101127000 101127010 101127020 101127030 101127040 101127050

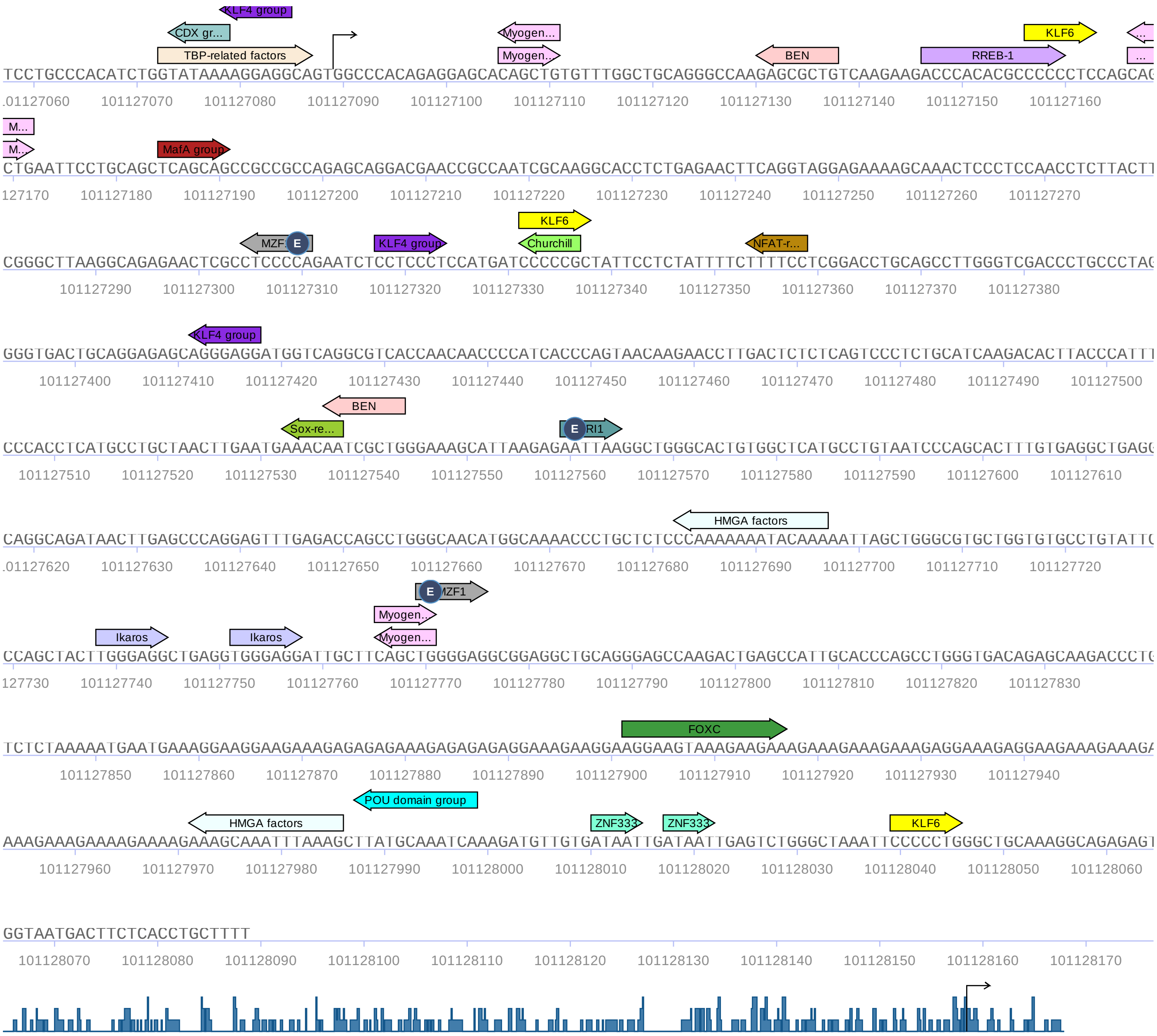
KLF4 group
CDX gr...
TBP-related factors
Myogen...
Myogen...
BEN
RREB-1
KLF6
...
TCCTGCCCACATCTGGTATAAAAGGAGGCAGTGGCCCACAGAGGAGCACAGCTGTGTTTGGCTGCAGGGCCAAGAGCGCTGTCAAGAAGACCCACACGCCCCCCTCCAGCAG
.01127060 101127070 101127080 101127090 101127100 101127110 101127120 101127130 101127140 101127150 101127160
M...
M...
MafA group
CTGAATTCCTGCAGCTCAGCAGCCGCCGCCAGAGCAGGACGAACCGCCAATCGCAAGGCACCTCTGAGAACTTCAGGTAGGAGAAAAGCAAACTCCCTCCAACCTCTTACTT
127170 101127180 101127190 101127200 101127210 101127220 101127230 101127240 101127250 101127260 101127270
KLF6
MZF1
E
KLF4 group
Churchill
NFAT-r...
CGGGCTTAAGGCAGAGAACTCGCCTCCCCAGAATCTCCTCCCTCCATGATCCCCCGCTATTCCTCTATTTTCTTTTCCTCGGACCTGCAGCCTTGGGTCGACCCTGCCCTAC
101127290 101127300 101127310 101127320 101127330 101127340 101127350 101127360 101127370 101127380
KLF4 group
GGGTGACTGCAGGAGAGCAGGGAGGATGGTCAGGCGTCACCAACAACCCCATCACCCAGTAACAAGAACCTTGACTCTCTCAGTCCCTCTGCATCAAGACACTTACCCATTT
101127400 101127410 101127420 101127430 101127440 101127450 101127460 101127470 101127480 101127490 101127500
BEN
Sox-re...
E
RI1
CCCACCTCATGCCTGCTAACTTGAATGAAACAATCGCTGGGAAAGCATTAAGAGAATTAAGGCTGGGCACTGTGGCTCATGCCTGTAATCCCAGCACTTTGTGAGGCTGAGG
101127510 101127520 101127530 101127540 101127550 101127560 101127570 101127580 101127590 101127600 101127610
HMGA factors
CAGGCAGATAACTTGAGCCCAGGAGTTTGAGACCAGCCTGGGCAACATGGCAAAACCCTGCTCTCCCAAAAAAATACAAAAATTAGCTGGGCGTGCTGGTGTGCCTGTATTC
.01127620 101127630 101127640 101127650 101127660 101127670 101127680 101127690 101127700 101127710 101127720
E
MZF1
Myogen...
Ikaros
Ikaros
Myogen...
CCAGCTACTTGGGAGGCTGAGGTGGGAGGATTGCTTCAGCTGGGGAGGCGGAGGCTGCAGGGAGCCAAGACTGAGCCATTGCACCCAGCCTGGGTGACAGAGCAAGACCCTC
127730 101127740 101127750 101127760 101127770 101127780 101127790 101127800 101127810 101127820 101127830
FOXC
TCTCTAAAAATGAATGAAAGGAAGGAAGAAAAGAGAGAGAAAGAGAGAGAGGAAAGAAGGAAGGAAGTAAAGAAGAAAGAAAGAAAGAAAGAGGAAAGAGGAAGAAAGAAAGA
101127850 101127860 101127870 101127880 101127890 101127900 101127910 101127920 101127930 101127940
POU domain group
HMGA factors
ZNF333
ZNF333
KLF6
AAAGAAAGAAAAGAAAAGAAAGCAAATTTAAAGCTTATGCAAATCAAAGATGTTGTGATAATTGATAATTGAGTCTGGGCTAAATTCCCCCTGGGCTGCAAAGGCAGAGAGT
101127960 101127970 101127980 101127990 101128000 101128010 101128020 101128030 101128040 101128050 101128060
GGTAATGACTTCTCACCTGCTTTT
101128070 101128080 101128090 101128100 101128110 101128120 101128130 101128140 101128150 101128160 101128170

Supplementary figure 5

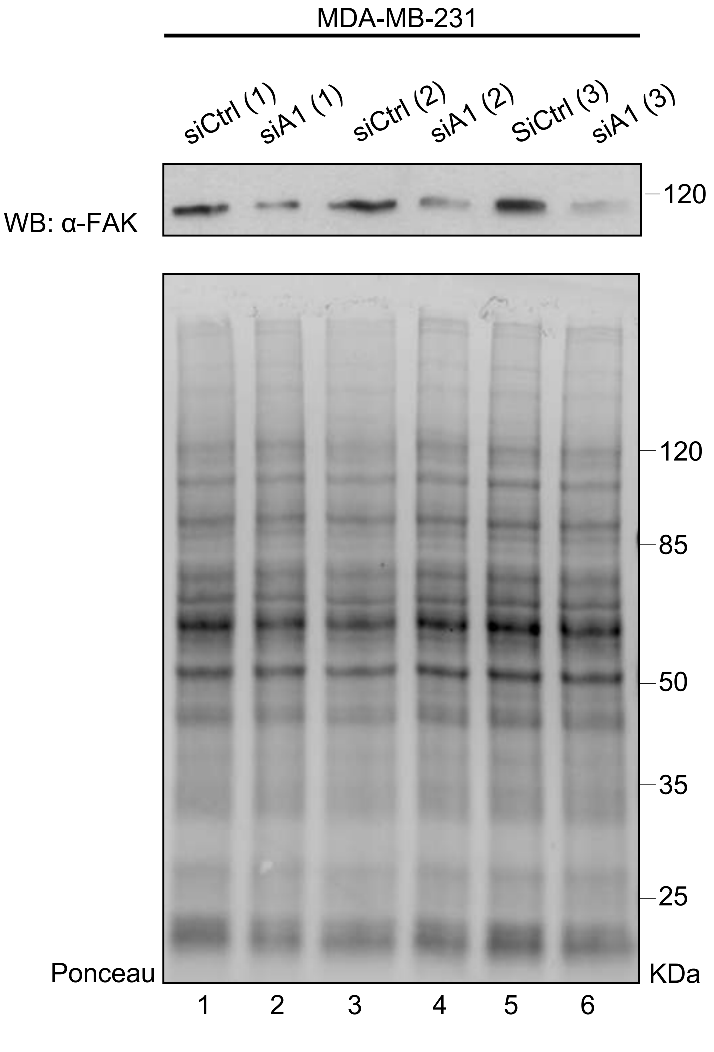

Supplementary Data – Methods

qRT–PCR primers	
**Primer Name**	**Oligo sequence (5' –> 3')**
NRP2 rev	CGATGTTCCCACAGTGTTTG
NRP2 for	CGAACCCAACCAGAAGATTG
CTSC rev	CCAGAATTGCCAAGGTCATC
CTSC for	TGTCAACTGCTCGGTTATGG
SLC1A5 rev	AAACCCACATCCTCCATCTC
SLC1A5 for	TCCGCTTCTTCAACTCCTTC
LGMN rev	GGAGTGGGATTGTCTTCAGAGT
LGMN for	GCAGGTTCAAATGGCTGGTAT
PLAUR rev	TGATGAGCCACAGGAAATGC
PLAUR for	TGGGTTAGACTTGTGCAACC
PLAU rev	AGCAAGGCAATGTCGTTGTG
PLAU for	GCTCAAGGCTTAACTCCAACAC
SERPINE1 rev	AAGGGGCAGCAATGAACATG
SERPINE1 for	TGGCTCAGACCAACAAGTTC
STC1 rev	TCCTTTGGAAAGTGGAGCAC
STC1 for	CTCAGGGAAAAGCATTCGTC
ADAM9 rev	AACATCTGGCTGACAGAACTGA
ADAM9 for	CTTGCTGCGAAGGAAGTACC
HMGA1 rev	AGCCCCTCTTCCCCACAAAGAGT
HMGA1 for	ACCAGCGCCAAATGTTCATCCTCA

siRNA sequences	
**siRNA name**	**siRNA sequence (5' –> 3')**
siNRP2	GCUUCUCUGCGCGUUACUATT
siLGMN	GCAUUGGUGCCGUUCCUAUTT
siA1_3	ACUGGAGAAGGAGGAAGAGTT
siCTRL	ACAGUCGCGUUUGCGACUGTT
